# Atypical *Legionella* GTPase effector hijacks host vesicular transport factor p115 to regulate host lipid droplet

**DOI:** 10.1126/sciadv.add7945

**Published:** 2022-12-16

**Authors:** Tao-Tao Chen, Yanling Lin, Shijun Zhang, Shuxin Liu, Lei Song, Wenhong Zhong, Zhao-Qing Luo, Aidong Han

**Affiliations:** ^1^ State Key Laboratory for Cellular Stress Biology, School of Life Sciences and Faculty of Medicine, Xiamen University, Xiamen, Fujian 361102, China.; ^2^ The Key Laboratory of Innate Immune Biology of Fujian Province, Provincial University Key Laboratory of Cellular Stress Response and Metabolic Regulation, Biomedical Research Center of South China, Key Laboratory of OptoElectronic Science and Technology for Medicine of the Ministry of Education, College of Life Sciences, Fujian Normal University, Fuzhou, China.; ^3^ Department of Respiratory Medicine and Center of Pathogen Biology and Infectious Diseases, Key Laboratory of Organ Regeneration and Transplantation of the Ministry of Education, State Key Laboratory of Zoonotic Diseases, The First Hospital, Jilin University, Changchun, China.; ^4^ Purdue Institute of Inflammation, Immunology and Infectious Disease, Department of Biological Sciences, Purdue University, West Lafayette, IN 47907, USA.

## Abstract

The intracellular bacterial pathogen *Legionella pneumophila* uses hundreds of effector proteins to manipulate multiple processes of the host cells to establish a replicative niche known as *Legionella*-containing vacuole (LCV). Biogenesis of the LCV has been known to depend on host small guanosine triphosphatases (GTPases), but whether bacterial effector GTPases are also involved remains unknown. Here, we show that an ankyrin repeat containing effector LegA15 localizes directly in host lipid droplets (LDs), leading to Golgi apparatus fragmentation of the host cells by hijacking the host vesicular transport factor p115. LegA15 is a GTPase with a unique catalytic mechanism, unlike any eukaryotic small GTPases. Moreover, the effector LegA15 co-opts p115 to modulate homeostasis of the host LDs in its GTPase-dependent manner. Together, our data reveal that an atypical GTPase effector regulates the host LDs through impeding the vesicle secretion system of the host cells for intracellular life cycle of *Legionella*.

## Teaser

Legionella effector LegA15 as a GTPase hijacks host p115 to regulate lipid droplets and subvert the host secretory pathway.

## INTRODUCTION

*Legionella pneumophila*, a Gram-negative bacterial pathogen, causes an atypical pneumonia known as legionnaires’ disease (*1*). The *Legionella* invades macrophages and generates a *Legionella*-containing vacuole (LCV), a unique compartment for bacterial replication to take place. The LCV is successively diverted away from the host endosomal-lysosomal degradation pathway as a result of the bacterial Dot/Icm type IV secretion system (T4SS), which translocates more than 300 effectors into the host cell (*2*, *3*). A number of them, containingeukaryotic-like domains (*4*), compete with the host cell protein counterparts and thus manipulate many important physiological processes of host cells.

Eukaryotic small guanosine triphosphatases (GTPases) are key regulators of intracellular membrane trafficking and the fusion with target membranes (*5*). In the past decade, *L. pneumophila* has been shown to hijack the host small GTPases by multiple mechanisms to modulate host vesicular transport pathways (*5*, *6*). A recent investigation on 80 *Legionella* genomes has shown that *Legionella* also encodes more than 180 effector proteins that contain a small GTPase–like domain (*7*). However, whether any of these effectors acts as a GTPase in modulation of host vesicle trafficking remains unknown.

*A Legionella* effector LegA15, also known as AnkD, is one of 11 ankyrin repeat–containing effectors in *L. pneumophila* (*8*). A recent crystal structure suggested that it is a cysteine protease (*9*); however, its function remained a mystery. Here, with crystal structures of LegA15 and its binary complex with a guanosine triphosphate (GTP) analog, we showed that LegA15 is, in fact, a GTPase. LegA15 localized directly in the host lipid droplets (LDs) and recruited the host vesicular transport factor p115, such that this effector induced fragmentation of the host Golgi apparatus, impairing the early secretory pathway of the host cells. We further showed that LegA15 modulates LD homeostasis of the host cells depending on its interaction with p115 and its GTPase activity. Together, our data revealed that LegA15 acts like a eukaryotic small GTPase to manipulate the host vesicle trafficking and secretory pathway for the intracellular life cycle of *Legionella*.

## RESULTS

### LegA15 targets the host LDs through its ankyrin repeats

**Fig. 1.**
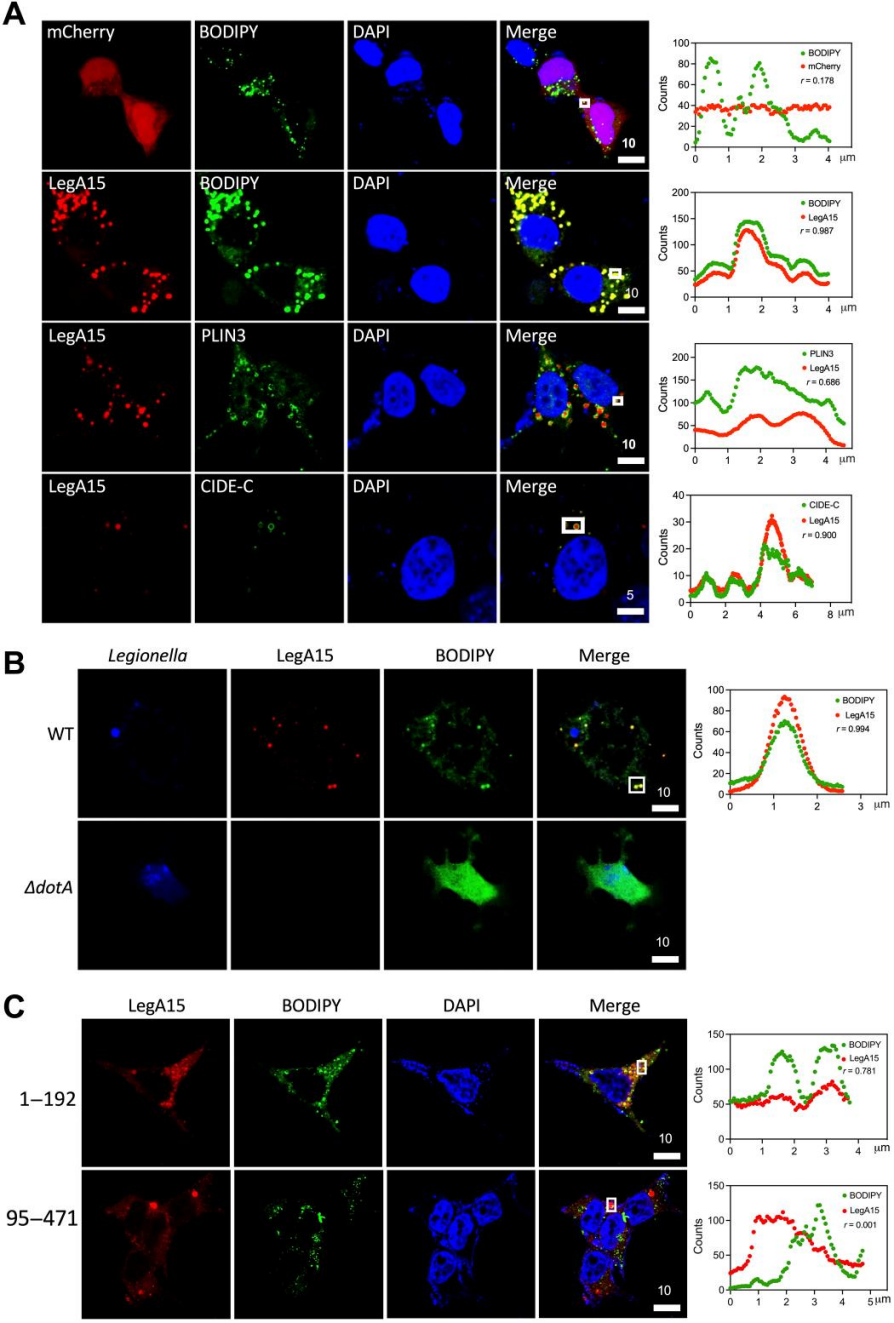
LegA15 targets host LDs. (**A**) Ectopically expressed LegA15-mCherry colocalizes with host LDs. Biomarkers PLIN3 and CIDE-C were shown in red by their antibodies. (**B**) LegA15 secreted by *L. pneumophila* colocalizes with the host LDs. The *L. pneumophila* WT and Δ*dotA* strains carrying a complemented LegA15 plasmid infected macrophage bone marrow–derived macrophage (BMDM) cells for 2 hours. Intracellular bacteria were detected with *Legionella*-specific antibody in blue. LegA15 was detected with the anti-LegA15 antibody in red. LDs were stained by the BODIPY dye in green. (**C**) The N-terminal domain of LegA15 is required for its LD localization. Two fragments of LegA15 in mCherry fusion were expressed in HEK293T cells shown in red. LDs were stained by the BODIPY dye in green, and nuclei were stained by 4′,6-diamidino-2-phenylindole (DAPI) in blue. Scale bars are in micrometer scale. Shown on the right were colocalization analyses with Pearson correlation (*r*) on region of interest (ROI) in white boxes. *X* axis is width of ROI and *y* axis is pixel counts for the fluorescence intensity of LegA15 (red curve) and host LDs (green curve).

The Dot/Icm effector LegA15, a 471–amino acid protein encoded by gene *lpg2456* of *L. pneumophila* Philadelphia, harbors an ankyrin repeat (ANK) domain that is known to mediate protein-protein interactions in eukaryotic cells (*10*, *11*). Ectopic expression of LegA15 in three mammalian cell lines,human embryonic kidney (HEK) 293T, L292, and HeLa, caused significant cell death (fig. S1A), suggesting that it targets one or more essential cellular processes. Unexpectedly, we did not observe its colocalization with several organelles, including Golgi, endoplasmic reticulum (ER), endosome, and mitochondria but with intracellular LDs (Fig. 1A and fig. S1B). Its colocalization to the LDs was further confirmed using two LD-specific protein markers perilipin-3 (PLIN3) and cell death–inducing DFF45-like effector C (CIDE-C) (*12*, *13*). Consistently, LegA15 was found to nicely colocalize with the host LDs in macrophage BMDM cells infected with *L. pneumophila* wild-type (WT) strain containing a LegA15–overexpressing plasmid (Fig. 1B).

Because LegA15 contains an ANK domain for protein-protein interactions, we then wondered whether this domain mediates the LD localization. We overexpressed two LegA15 fragments (amino acids 1 to 192 and 95 to 471) containing its ANK domain and C-terminal unknown domain, respectively, in HEK293T cells, and found that the ANK domain but not the C terminus was colocalized with the LDs (Fig. 1C). Together, these data suggested that LegA15 is directly associated with the host LDs through its ankyrin repeats.

### LegA15 interacts with the vesicular transport factor p115

**Fig. 2.**
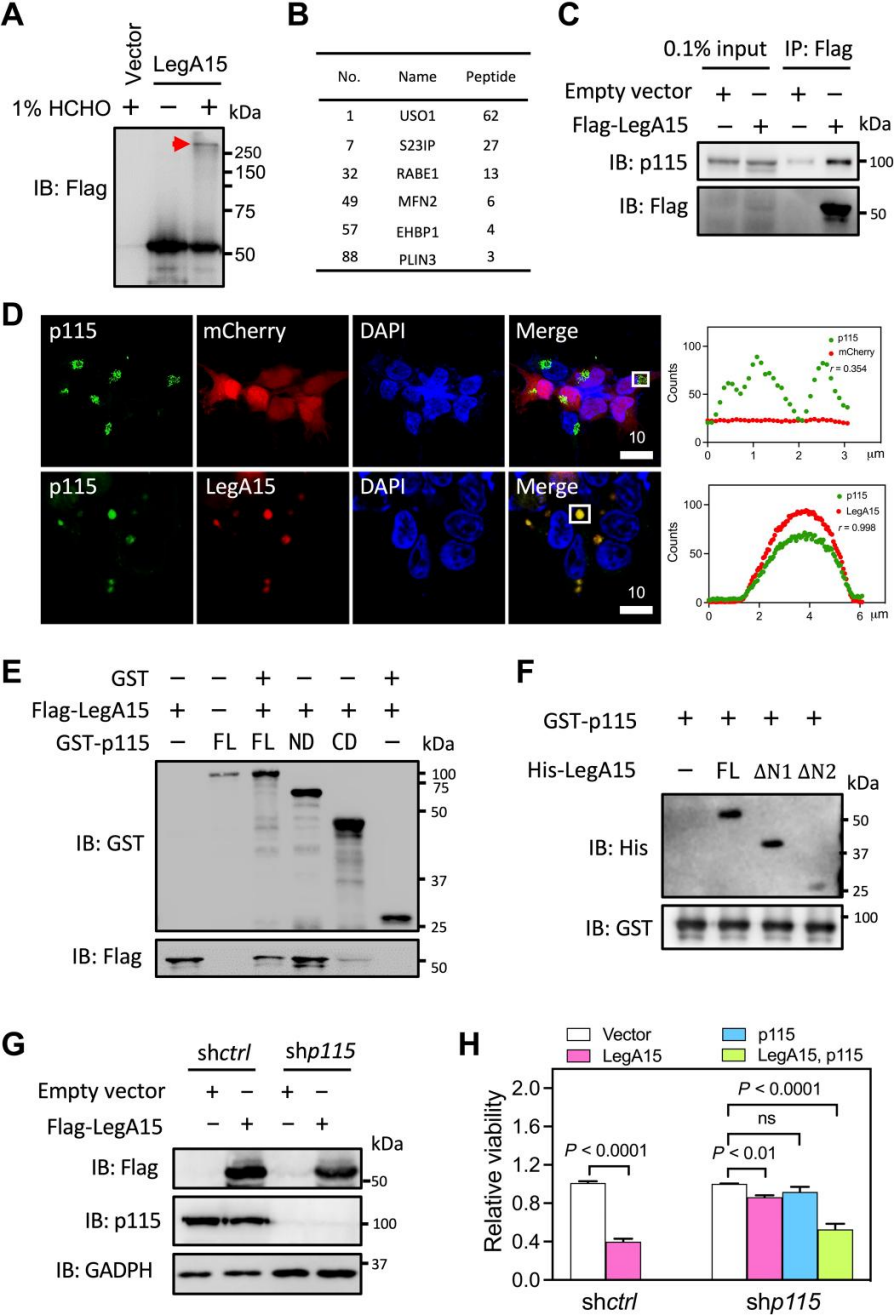
LegA15 interacts with host vesicular transport factor p115. (**A**) Immunoblotting (IB) analysis of the IP fraction. A cross-linked protein complex was positioned by a red arrow. (**B**) Putative LegA15 interaction proteins. A full list of proteins in vesicle trafficking and membrane fusion are table S1. (**C**) LegA15 interacts with endogenous p115. LegA15 was expressed in HEK293T and immunoprecipitated with anti-Flag antibody, and p115 was detected by anti-p115 antibody. (**D**) LegA15 colocalizes with p115 in the HEK293T cells expressing LegA15-mCherry. p115 in green was stained with anti-p115 antibody. Nuclei were in blue. Scale bars are in micrometer scale. Shown on the right were colocalization analyses with Pearson correlation (*r*) on ROI in white boxes as Fig. 1. (**E**) The N-terminal domain of p115 interacts with LegA15 in a GST pulldown experiment. GST-p115 fusion proteins [the full-length, ND (1 to 651) and CD (652 to 964)] were used to pull-down Flag-LegA15, and detected by anti-Flag antibody. Protein loading was examined by anti-GST antibody. (**F**) His-LegA15 [the full-length, ΔΝ1 (95 to 471) and ΔΝ2 (195 to 471)], GST-p115 (1 to 651) was pulled down and detected by anti-His antibody with protein loadings by anti-GST antibody. (**G**) *p115*-knockdown HEK293T cell line expressing LegA15. Flag-LegA15 was expressed for 16 hours, and detected by p115 and Flag antibodies. GAPDH, glyceraldehyde-3-phosphate dehydrogenase. (**H**) Knockdown of *p115* suppresses the cytotoxicity of LegA15 after transfection for 36 hours. Transfection with its empty vector was a negative control. Shown is relative viability from three independent experiments and analyzed by one-way analysis of variance (ANOVA) assay. ns, not significant.

We next sought to identify host proteins that interact with LegA15. We expressed Flag-LegA15 in HEK293T cells and treated the cells with formaldehyde and performed an immunoprecipitation (IP) using the anti-Flag antibody, followed by mass spectrometric analysis (Fig. 2A). More than 1000 potential LegA15 binding proteins were found in the co-IP fractions, and nearly 100 proteins were associated with vesicle trafficking and membrane fusion (table S1). A yeast USO1 homolog p115 was found on the top of the list with 62 unique peptides (Fig. 2B). We further verified their interaction using anti-p115 antibody, showing that endogenous p115 was substantially enriched with LegA15 in the co-IP fraction of HEK293T cells expressing Flag-LegA15 (Fig. 2C), while other proteins were not readily detectable, including two LD-associated proteins PLIN3 and CIDE-C (fig. S2). Consistently, LegA15 colocalized with p115 in the cells (Fig. 2D). Coexpressed LegA15 and p115 were also colocalized in HEK293T cells (fig. S3). These data therefore indicated that the effector LegA15 interacts with the host membrane tethering factor p115.

The p115 protein has a globular head (residues 1 to 651, ARM domain), a coiled-coil region, and a highly acidic C-terminal tail. To map their interaction regions, we produced proteins of two separate regions 1 to 651 (ND) and 651 to 962 (CD) of p115 in addition to its full length for a glutathione *S*-transferase (GST) pulldown experiment. The ND but not the CD strongly interacted with LegA15 (Fig. 2E), suggesting that the N-terminal ARM domain is dominant in the LegA15 binding. We further mapped the interaction domain of LegA15. Two ankyrin repeat deletions (ΔΝ1, 95 to 471 and ΔΝ2, 195 to 471) of LegA15 were able to interact with p115 and its full-length protein, indicating that ankyrin repeats are dispensable for their interaction (Fig. 2F).

### Silencing *p115* suppresses the cytotoxicity of LegA15

Because ectopic expression of LegA15 was toxic to mammalian cells, we next asked whether its cytotoxicity depends on p115. We established a stable line from HEK293T cells with its p115 substantially knocked down by short hairpin RNA (shRNA) and further transiently expressed LegA15 in this cell line (Fig. 2G). Although silencing *p115* interfered with cell proliferation, we found that ectopic expression of LegA15 in this cell line did not induce cell death as seriously as it did in WT cells (Fig. 2H). Approximately 85% of the *p115* knockdown cells survived for 24 hours upon the expression of LegA15, but significantly lower when these cells were further complemented with p115. Notably, we did not observe any obvious changes of LDs in the *p115* knockdown cells (fig. S4). This experiment thus suggested that the cytotoxicity of LegA15 is dependent on p115.

### LegA15 induces Golgi fragmentation of the host cell

**Fig. 3.**
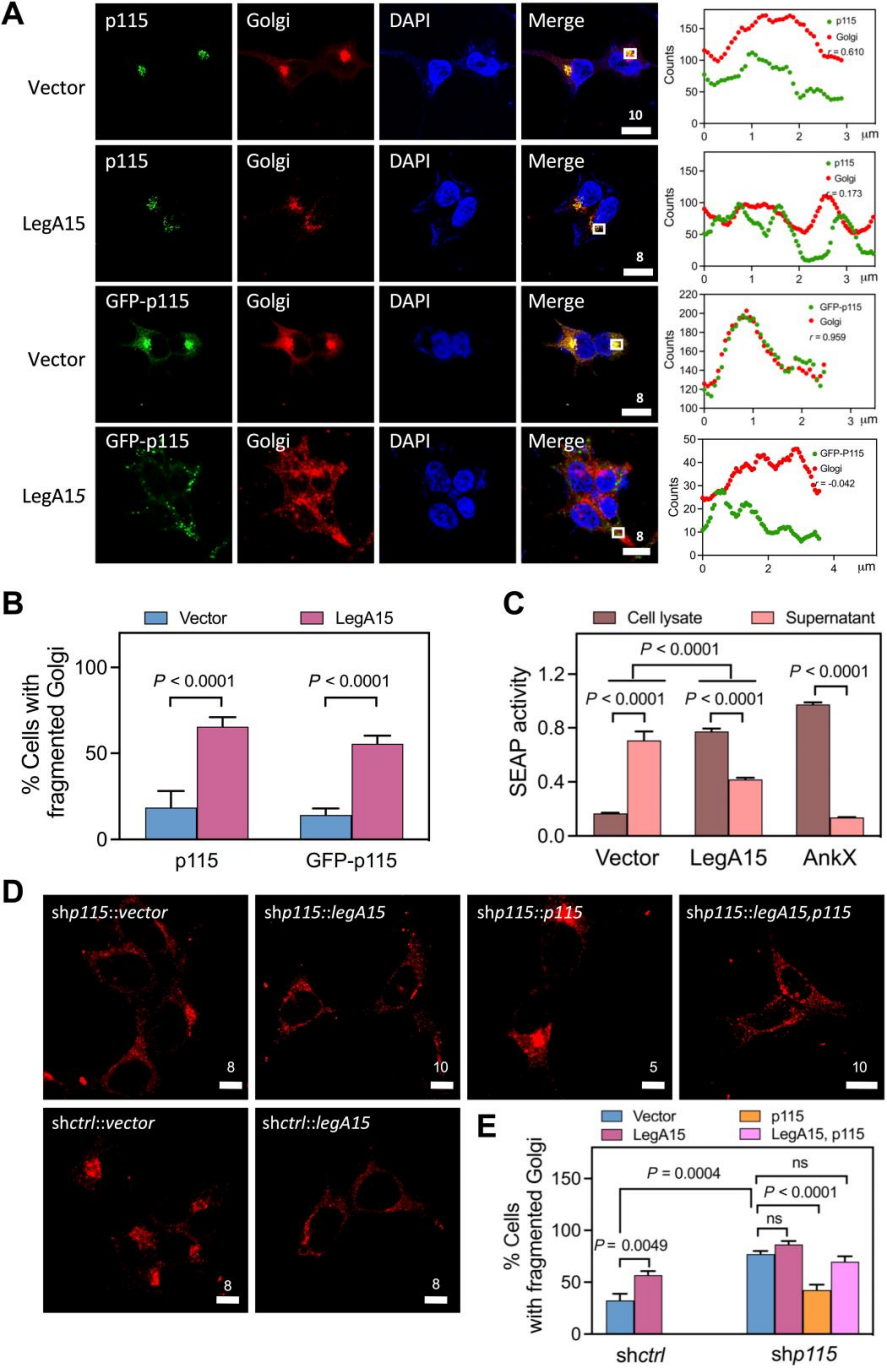
LegA15 induces fragmentation of the host Golgi apparatus. (**A**) Golgi apparatus localization of p115 is affected by overexpressed Flag-LegA15. GFP-p115 and endogenous p115 were detected by anti-p115 antibody in green. Golgi apparatus was stained with anti-syntaxin 6 antibody in red. Nuclei were in blue. Scale bars are in micrometer scale. Shown on the right were colocalization analyses with Pearson correlation (*r*) on ROI in white boxes as Fig. 1. Scale bars are in micrometer scale. (**B**) LegA15 induces fragmentation of Golgi apparatus in host cells. The quantification of (A) was calculated for three groups using more than 100 randomly selected cells as each group. The significance was calculated by *t* test. (**C**) LegA15 inhibits the secretion of SEAP. The HEK293T cells coexpressed with Flag-LegA15 and SEAP for 16 hours were used to measure the SEAP activity in culture medium and cell lysates, separately. Transfection with its empty vector was used as a negative control. In addition, *Legionella* effector AnkX was used as a positive control. Quantitation shown was from three independent experiments and analyzed by one-way ANOVA assay. (**D** and **E**) The host p115 is required for LegA15 induced Golgi fragmentation. LegA15 was overexpressed in the HEK293T cells with p115 knockdown and complemented p115. The Golgi apparatus was stained with anti-syntaxin 6 antibody (red). Nuclei were in blue. Transfection with an empty vector was a negative control. The quantification of Golgi fragmentation (E) was calculated for three groups using more than 100 randomly selected cells as one group. The significance was calculated by *t* test.

The p115 protein as a biomarker for Golgi plays a key role in vesicular transport between the ER and the Golgi apparatus by tethering the coat-protein complex I (COPI) (*14*–*16*). We then wanted to know whether LegA15 disrupted function of Golgi by the interaction with p115. Both endogenous and overexpressed p115 was found highly enriched in Golgi (Fig. 3A). In presence of LegA15, however, p115 was drifted away from Golgi, leading to significant fragmentation of the Golgi apparatus (Fig. 3B). We further examined the functionality of the Golgi using secreted embryonic alkaline phosphatase (SEAP) as a reporter. Overexpression of LegA15 strongly inhibited the secretion of SEAP into culture medium comparably with a *Legionella* effector AnkX (Fig. 3C). However, overexpression of AnkX did not cause p115 dislocation or Golgi fragmentation (fig. S5), consistent with the fact that AnkX has been shown to disrupt the host cell secretion system through a covalent phosphocholination of Rab GTPases (*17*).

Last, we asked whether p115 was essential for LegA15-induced Golgi fragmentation. We overexpressed LegA15 in the *p115* knockdown cell line built above (Fig. 3, D and E). Comparing with the mock knockdown cells using scrambled shRNA, we did not observe a significant Golgi fragmentation. One possibility was that the *p115* knockdown excessively disintegrated Golgi as previously shown by Radulescu *et al.* (*18*). Complementation of p115 partially restored Golgi apparatus, but the Golgi became significantly fragmented in the cells with overexpressed LegA15. Together, these data demonstrated that the effector LegA15 directly interacts with the host general vesicular transport factor p115, resulting in the fragmentation of Golgi apparatus and impaired vesicle trafficking in the host cells.

### LegA15 is a GTP-binding protein

**Fig. 4.**
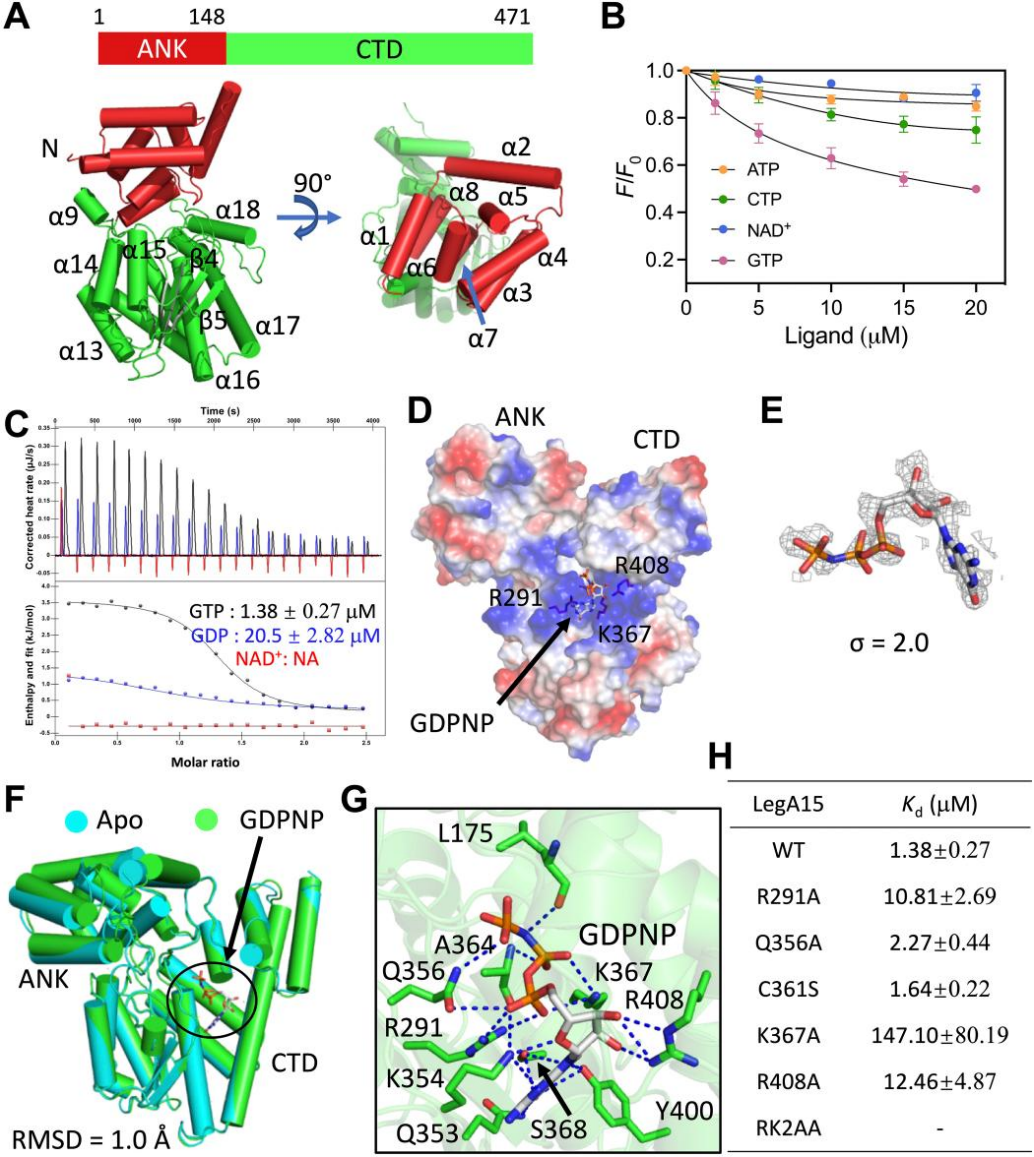
LegA15 is a GTP-binding protein. (**A**) A crystal structure of LegA15. Schematic diagram for protein domains is shown on the top, and a crystal structure is shown in a cartoon below. The N-terminalANK domain is in red, and the CTD is in green. The secondary structures are labeled. (**B**) Ligand binding analyses of LegA15 by synchronous fluorescence titration. LegA15 at 10 μM was used to titrate with 20 mM nucleotides. *Y* axis indicates relative fluorescence intensity of LegA15 to their initial intensity without ligands. (**C**) The GTP/GDP binding was analyzed by ITC. GTP/GDP/NAD^+^ are colored in black, blue, and red, respectively. Titration profiles are shown on the top, and fittings below. (**D**) A complex structure of LegA15 with a GTP analog GDPNP shown as a surface model colored according to the electrostatic surface potential [contoured from −5 *k*_B_*T* (red) to +5 *k*_B_*T* (blue)]. Residues in GDPNP binding are shown as stick models. (**E**) The simulated annealing omit map of GDPNP contoured at 2σ. The map in gray mesh is fitted with the ligand in a stick model. (**F**) Structural comparison of Apo-LegA15 (cyan) with its GDPNP complex (green). The difference is indicated by RMSD. (**G**) The detailed interaction of GDPNP in the binding pocket of LegA15. Stick models are shown for GDPNP in gray and amino acids in green important for the binding. Hydrogen bonds are highlighted in the blue dashed lines. (**H**) Mutations of key amino acids disrupted the LegA15 binding with GTP in ITC. All titration profiles are shown in fig. S7.

To further understand the molecular mechanism of LegA15, we solved its crystal structure at a resolution of 2.59 Å (table S2). LegA15 contains an N-terminal ANK and a C-terminal domain (CTD) (Fig. 4A). The CTD consists of a β-strand lobe sandwiched by two α-helical clusters. Structural similarity search using this CTD in PDBeFold online suggested that several nucleotide binding proteins are structurally similar to LegA15, even though their sequence identities are less than 10% (table S3). We then explored the nucleotide binding possibilities by fluorescence titration experiment, which showed that LegA15 was able to bind GTP but not adenosine triphosphate (ATP), cytidine triphosphate (CTP), or nicotinamide adenine dinucleotide (NAD^+^) (Fig. 4B and fig. S6). Isothermal titration calorimetry (ITC) experiment further confirmed that LegA15 bound both GTP and guanosine diphosphate (GDP) but not NAD^+^ (Fig. 4C). The disassociation constants (*K*_d_) for binding by GTP and GDP are 1.38 and 20.5 μM, respectively.

We next solved a binary structure of LegA15 bound with an unhydrolyzable GTP analog 5′-guanylyl imidodiphosphate (GDPNP) at a resolution of 2.27 Å (Fig. 4D and table S2). GDPNP was well defined by its electron density (Fig. 4E). GDPNP is shown to bind a positive-charged pocket formed by Arg^291^, Arg^408^, and Lys^367^ without inducing a large conformational change of LegA15 because LegA15 in this complex is well aligned to its ligand-free form [root mean square deviation (RMSD) of 1.0 Å] (Fig. 4F). GDPNP also makes additional interactions with LegA15 (Fig. 4G). The guanosine hydrogen bonds with residues of Gln^353^, Lys^354^, Ser^368^, and Tyr^400^ in addition to Lys^367^. The γ-phosphate of GTP is anchored by Ala^364^ and Gln^356^ via hydrogen bonds, while its β-phosphate hydrogen bonds with both Arg^291^, Gln^356^, and Lys^367^. The α-phosphate hydrogen bonds with Lys^367^. We then evaluated the mutational effect of these key residues by ITC experiments (Fig. 4H and fig. S7). The alanine substitutions for Arg^291^, Lys^367^, and Arg^408^ severely impaired its GTP binding capacity, but Q356A mutation was less effective. A RK2AA (triple mutant R291A/K367A/R408A) mutant combined with Arg^291^, Arg^408^, and Lys^367^ as well as the K367A mutant almost abolished the GTP binding. Together, these data strongly supported that LegA15 is a GTP-binding protein.

### LegA15 is a GTPase

**Fig. 5.**
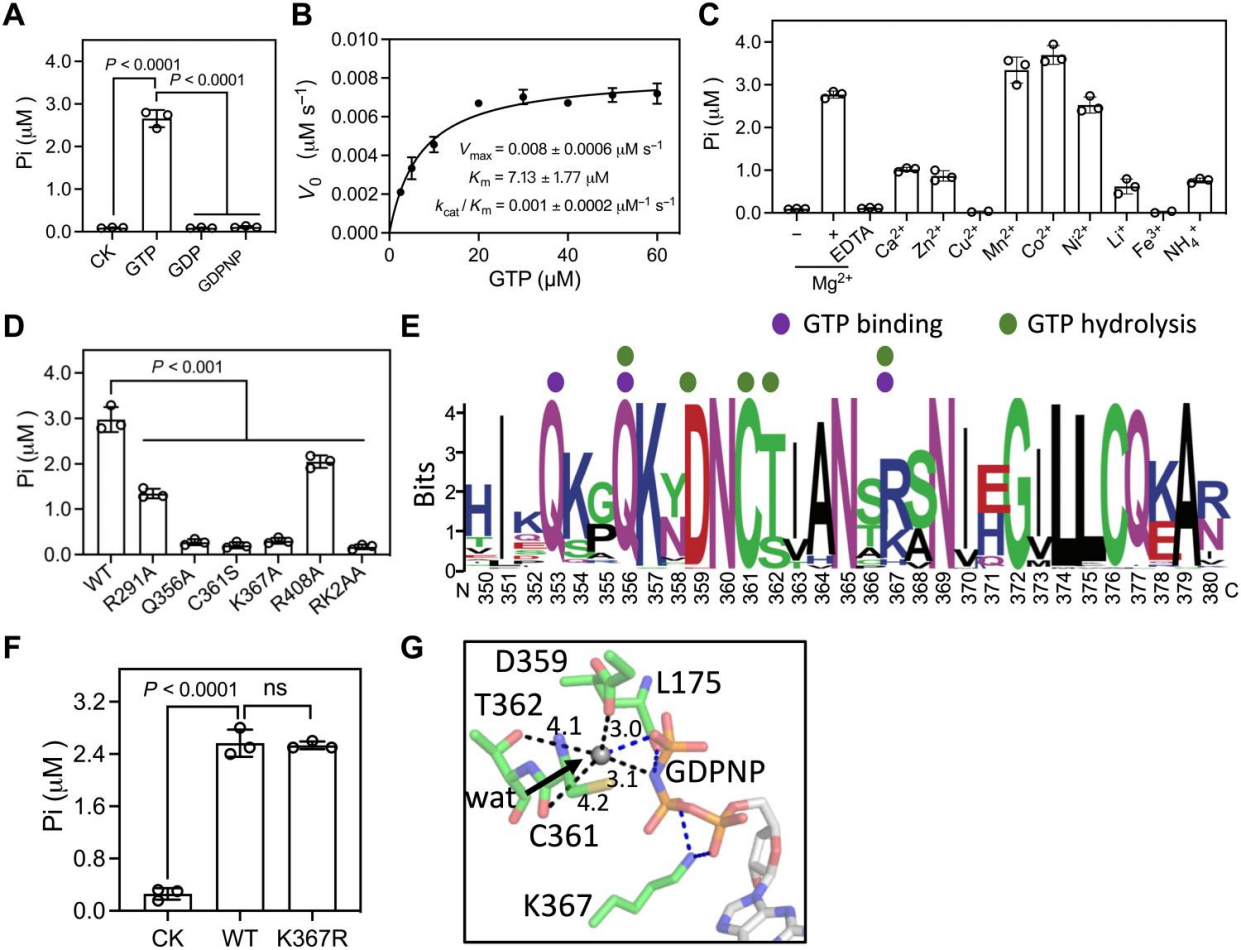
LegA15 has GTPase activity. (**A**) The GTPase activity of LegA15. The activity was measured by free phosphate production in the reaction using a colorimetric ATPase/GTPase assay kit. The reaction without the protein was used as a negative control (CK). (**B**) Enzyme kinetics of LegA15 measured using a concentration gradient of GTP with 1 μM LegA15 on a 96-well plate. The steady-state kinetic parameters were derived by fitting to the Michaelis-Menten equation. (**C**) The divalent metal ions were required for the GTPase activity. GTP was preloaded into recombinant LegA15 protein and exchanged to reaction buffers with additional ions. (**D**) The GTPase activity of LegA15 was impaired by all GTP binding mutations. (**E**) Conservation analysis of residues implicated in GTP binding pocket from 39 *Legionella* LegA15 homologs. Residues are colored by WebLogo (http://weblogo.berkeley.edu/) with key residues in GTP binding and hydrolysis indicated by colored dots. (**F**) Replacement of Lys^367^ with arginine preserved the GTPase activity of LegA15. The reaction without the protein was used as a negative control (CK). (**G**) The active site of LegA15 with a nucleophilic water molecule. Residues and GDPNP are shown in stick models with water in gray sphere and the distances (Å) in black dashed lines. Four hydrogen bonds are in blue dashed lines.

GTPases play their roles in cellular signaling by alternating in their GTP- and GDP-bound forms (*19*, *20*). The finding that LegA15 binds GTP and GDP with different affinity prompted us to test whether LegA15 hydrolyses GTP. We found that LegA15 efficiently hydrolyzed GTP, but not GDP and GDPNP, similar to eukaryotic small GTPases (Fig. 5A). The *kcat*/Km was about 1.0 × 10^−3^ μM^−1^ s^−1^ and the maximum velocity (*V*_max_) was about 8.0 × 10^−3^ μM s^−1^ for LegA15 on GTP (Fig. 5B). The reaction required divalent ions, such as Mg^2+^, Mn^2+^, Co^2+^, and Ni^2+^ (Fig. 5C). These results indicated that LegA15 has a GTPase activity in dependence on several divalent metal ions.

Aided by the crystal structure of LegA15 and GDPNP, we then determined whether those implicit residues for its GTP binding affected its GTPase activity. The Q356A, K367A, and RK2AA mutants nearly lost the GTPase activity, while R291A and R408A mutants had significantly reduced the activity (Fig. 5D). In addition, the cytotoxicity of the overexpressed LegA15 with these mutations was diminished, and their ability of inhibiting cellular secretion was also severely impaired (fig. S8).

We further aligned 61 unique *Legionella* LegA15 protein sequences with their identity as low as 42% and found that the most conserved motif at residues 350 to 380, where Gln^353^, Gln^356^, Asp^359^, and Cys^361^ are highly conserved (Fig. 5E). Lys^367^, one of the most important residues, however, is replaced by arginine in most *Legionella* species. The substitution by arginine barely affected the GTPase activity of LegA15 (Fig. 5F). In addition, highly conserved residues Cys^361^ and Thr^362^ possibly interact with the γ-phosphate of GDPNP through an intermediate water, which is in a good position for a nucleophilic attack (Fig. 5G). Consistently, we observed that the C361S mutation retained the GTP binding but abolished the GTPase activity (Figs. 4H and 5D). In addition, its cell toxicity and blockage to the host cell SEAP activity were significantly reduced (fig. S8). A previous structure proposed a putative catalytic triad consisting of H268-N290-C361 (fig. S9A) (*9*). However, while the N290A mutant showed a mild effect, the H268A mutant had not much effect when compared with other mutations shown above (fig. S9, B and C).

Together, these structures and biochemical experiments demonstrated that LegA15 is a GTPase with a unique enzymatic mechanism that Lys367 stabilizes GTP at its transition state, and Cys^361^ activates a water molecule for in-line nucleophilic attack to the γ-phosphate, leading to a GTP hydrolysis (Fig. 5G). In contrast, canonical GTPase activation proteins, such as RasGAPs, ArfGAPs, RhoGAPs, and RabGAPs, often harbor an arginine and a negative residue Gln/His/Asn for catalysis (fig. S9D) (*21*).

### LegA15 co-opts p115 for the regulation of host LDs

**Fig. 6.**
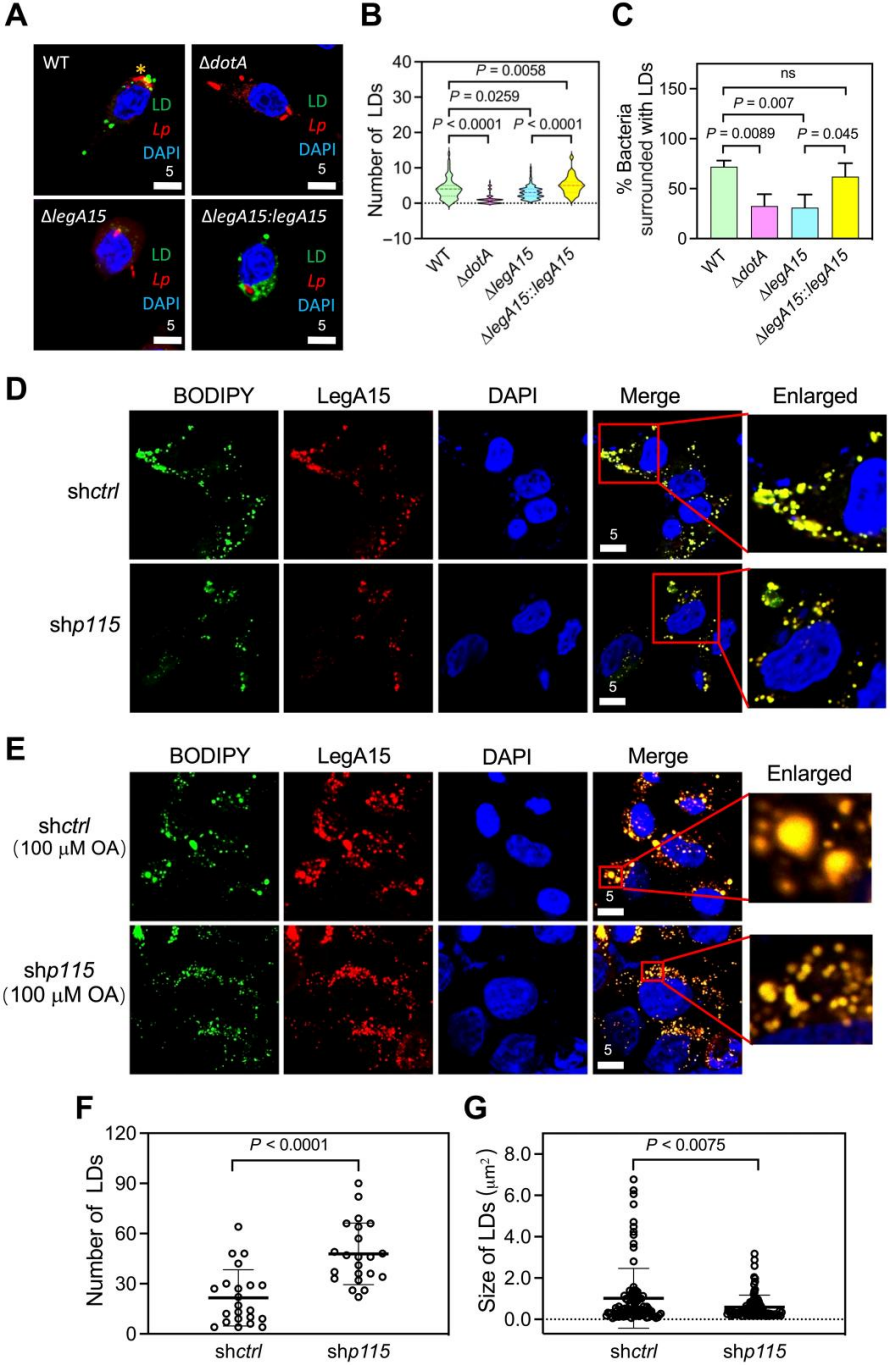
The effects of LegA15 are dependent on p115. (**A** and **B**) *L. pneumophila* infection led to LD accumulation of host cells. The Raw264.7 cells were infected by *L. pneumophila* at 0.1 multiplicity of infection for 12 hours and stained with *Legionella*-specific antibody in red. LDs were in green, and nuclei were in blue. Quantification of LD numbers (B) was calculated using more than 100 cells containing bacteria in ImageJ and analyzed by one-way ANOVA assay. (**C**) LegA15 promoted host LDs to LCV. *Y* axis represents a percent ratio of bacteria with LD at a close distance of less than 1 μm [yellow * in (A)]. The ratio was averaged of three times with more than 100 intracellular bacteria counted each time and analyzed by one-way ANOVA assay. (**D** to **G**) Intracellular LDs affected by the *p115* knockdown. LegA15-mCherry shown in red was overexpressed in the *p115* knockdown cells (D) and further treated with 100 μM sodium oleate (OA) for 16 hours (E). The number (F) and size (G) of LDs in 50 cells in (D) were measured in ImageJ and analyzed by one-way ANOVA assay. LDs were in green. Scale bars are in micrometer scale.

We next wanted to explore physiological implications for LegA15 that localizes in host cell LDs. We first created a *legA15*-deleted *L. pneumophila* strain (fig. S10A). However, the deletion did not affect bacterial growth in mouse BMDM cells (fig. S10B). Consistently, LegA15 was previously shown not important for the bacterium to grow in *Drosophila* cells (*22*). The number of host LDs significantly increased when infected by *L. pneumophila* WT but not by its *dotA* mutant that is defective in the Dot/Icm secretion system (Fig. 6A). The *legA15* deletion strain induced less amount of host LDs, and a bacterial strain with complemented LegA15 induced a larger amount of host LDs. Moreover, we found that the LDs were significantly accumulated around the LCVs (Fig. 6C).

We then asked whether the LD regulation by LegA15 depended on the host protein p115. We analyzed the localization of LegA15 in the *p115*-silenced cells. Notably, LegA15 was not required for *L. pneumophila* to grow in the host cells, which was neither affected by the p115 depletion (fig. S10, C and D). The overexpressed LegA15 was still associated with LDs, but their sizes seemed smaller in the *p115* depletion cells (Fig. 6D). To quantify such an effect, we treated the cells with 100 μM sodium oleate, a reagent that promotes LD biogenesis (Fig. 6E). We observed that ectopic expression of LegA15 significantly increased the number of LDs but resulted in smaller sizes in the p115-depleted cells (Fig. 6, F and G). These data suggested that *Legionella* secretes the effector LegA15 to modulate host LDs via hijacking the host general vesicular transporter p115.

### LegA15 regulates the host LD homeostasis in its GTPase-dependent manner

**Fig. 7.**
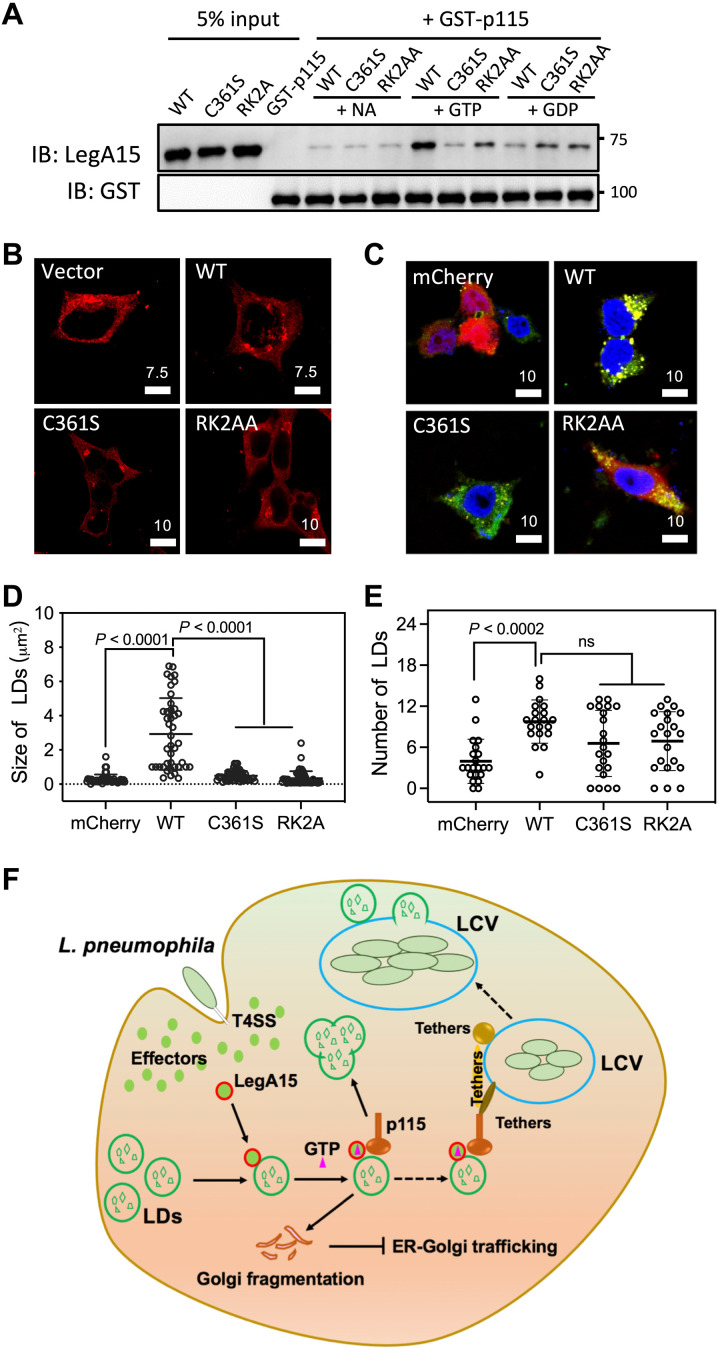
GTPase activity of LegA15 is essential for its function in host cells. (**A**) GTP binding facilitates the interaction between LegA15 and p115. The GST-p115 and LegA15 interaction was analyzed by a GST pulldown experiment with GTP or GDP in the binding buffer. GST-p115 and LegA15 proteins retained on GST beads were detected by GST- and LegA15-specific antibodies, respectively. (**B**) The GTPase activity of LegA15 is required for fragmentation of Golgi apparatus. Cells overexpressing LegA15 or its GTPase defective mutants were stained with anti-p115 antibody in red. Scale bars are in micrometer scale. (**C** to **E**) The GTPase activity of LegA15 is required for modulation of LDs. The LegA15-mCherry or its mutants were overexpressed in HEK293T cells (C), and the size (D) and number (E) of LDs were quantified in ImageJ. LDs were in green, and nuclei were in blue. Scale bars are in micrometer scale. The size and number of LDs were averaged from 50 cells and analyzed by one-way ANOVA assay. (**F**) A model for *L. pneumophila* infection, which releases an effector LegA15 to regulate host LDs by hijacking a general vesicular transporter p115.

In mammalian cells, active small GTPase of Rab1 recruits p115 for vesicle trafficking and fusion (*23*). The GTPase activity of LegA15 prompted us to wonder whether it also affects the LegA15 and p115 interaction. We first tested this by a GST pulldown assay using a fusion protein GST-p115 (1 to 651), a dominant domain that binds LegA15 (Fig. 7A). LegA15 was retained more strongly in the presence of GTP, compared to the same condition supplemented with GDP or without GTP. A GTP-binding mutant RK2AA and a GTPase-defective mutant C361S significantly reduced their affinity to p115. Consistently, the C361S and RK2AA mutants were less effective in the fragmentation of host Golgi (Fig. 7B). Moreover, the expression of mutants RK2AA and C361S did not lead to the formation of LD clusters as seen with WT protein, although both still slightly increased the LD abundance (Fig. 7, C to E). Together, these data suggested that LegA15 acts like a eukaryotic small GTPase to recruit the host vesicle fusion protein p115 for regulation of the host LDs, leading to loss of p115 in Golgi, thus inducing Golgi fragmentation.

## DISCUSSION

*L. pneumophilla* promotes the biogenesis of the replication-permissive LCV in host cells by its large cohort of effectors. The host small GTPases are among the targets of these effectors for *Legionella* to capture and selectively incorporate host vesicles into the bacterial phagosome (*24*, *25*). Here, we show that a *Legionella* effector LegA15 is a GTPase with high similarity to eukaryotic small GTPases in many aspects.

### LegA15 functions as an atypical GTPase effector

The past decade has been seen that *L. pneumohpella* exploits host small GTPases through a variety of effectors, which are often functioning as guanosine nucleotide exchange factors (GEFs) or GTPase-activating proteins (GAPs) to regulate host small GTPases, such as RalF (*26*) and LepB (*27*). Some effectors, such as AnkX (*28*) and SidE family (*29*, *30*), directly modify the host small GTPases. Some effectors, such as LidA, directly bind the host small GTPase and interfere with early secretory vesicular trafficking (*31*). However, *Legionella* encode about 180 proteins with a small GTPase-like domain, some of which are classified as Rho- and Rab-like proteins (*7*). LegA15 bears a GTPase activity without GEFs or GAPs, which is distinct from the conventional small GTPases (Fig. 5). However, similar to the conventional small GTPases, LegA15 interacts with p115 in GTP- and GTPase-dependent manner for regulation of the host LDs (Figs. 6 and 7).

It has become more and more indisputable that LDs are not just a major storage of cellular lipids, but a dynamic organelle associated with a number of proteins, including many small GTPases (*32*, *33*). Rab18 and Rab40c have been shown in LDs, which regulate the LD biogenesis (*34*, *35*). Here, we show that a *Legionella* effector LegA15, including both WT and its GTPase defective mutants, is localized in host LDs (Figs. 1 and 7). However, only its WT but not these mutants, when overexpressed, significantly increased the size and number of host LDs because these mutants lost their interaction with the host tethering protein p115 and/or the regulation by GTP/GDP (Fig. 7). It, however, remains to be investigated whether LegA15 competes with host small GTPases for host tethering proteins because no small GTPases were found in our cross-linked IP fractions (table S1). In addition, we found that LegA15 stand-alone is catalytically active (Fig. 5), but whether it has its specific GEFs and GAPs remains further investigation.

### *L. pneumophila* manipulates the host p115 in membrane fusion

The tethering protein p115 is required for the early secretion and fusion of ER-derived COPII vesicle to Golgi apparatus (*23*, *36*). The successive vesicle tethering, docking, and fusion in Golgi appatatus are required for maintenance of Golgi integrity. Continued budding of vesicles without sufficient membrane fusion leads to fragmentation of the Golgi apparatus (*37*). p115 plays a key role in consecutive linkages, joining the long tethers, such as Giantin and GM130, and the short tethers, such as GOS-28 and syntaxin-5, as part of cognate SNAREpin assembly, to facilitate membrane fusion and maintain the Golgi architecture (*38*, *39*). Depletion of p115 or abrogation of its tethers would induce Golgi fragmentation (*40*). Given that LegA15 induces the Golgi fragmentation through p115 (Figs. 2 and 3), it is a good possibility that dislocation of host p115 from Golgi to LDs by LegA15 disrupts assemblies of the SNARE complex and/or Golgi tethering complex, thereby inducing Golgi apparatus fragmentation in host cells (Fig. 7F).

Furthermore, we showed that *L. pneumophila* infection significantly induces the number of host LDs that are increasingly accumulated around LCVs (Fig. 6). Many tethering factors, such as GRASP55, Sec22b, syntaxins, and SNARE proteins (*41*), have been shown accumulated on LCV (*42*–*47*). The N-terminal AMR domain of p115 targets COPII vesicle membrane through binding Rab1 (*48*), and the acidic C terminus interacts with the cis-Golgi tethering complex (*49*). LegA15 was shown to interact with the AMR domain of p115 (Fig. 2E). Therefore, it is plausible to propose that LegA15 acts like a eukaryotic small GTPase and further recruits p115 to tether the host LDs onto LCV, such that *L. pneumophila* manipulates the host LDs for its LCV biogenesis (Fig. 7F). In addition, *L. pneumophila* also recruits and activates the host small GTPase Rab1 on LCV by a GEF effector SidM to promote the LCV biogenesis (*50*).

### Lipid droplets may serve as materials for *L. pneumophila* replication

The bacterial pathogens have been shown to target the host LDs as lipid and energy sources to facilitate pathogenic survival in host (*51*). Some bacteria produce foamy macrophages characterized by accumulated host LDs. *Mycobacterium tuberculosis* recruits LDs onto its phagosomes to exploit free fatty acid released from triacylglycerol as its major energy and carbon source (*52*, *53*). *Salmonella enterica* Typhimurium makes use of host LDs as a membrane source of *Salmonella*-containing vacuole (*54*). Similarly, we showed that the host LDs are significantly gathered up around the LCVs (Fig. 6). Therefore, *L. pneumophila* may likely use the host LDs as the membrane or nutrition source for its intracellular life cycle, in agreement with the general functions of LDs (*55*).

## MATERIALS AND METHODS

### Bacterial strains

All *L. pneumophila* strains were derived of the Philadelphia-1 strain Lp02 (*56*). The bacteria were grown on charcoal-yeast extract (CYE) plate or in *N*-(2-acetamido) 2-aminoethanesulfonic acid (ACES)–buffered yeast extract (AYE) broth with additional thymidine (200 μg/ml) for the thymidine auxotrophic mutant and its derivatives.

*Escherichia coli* strains DH5α and BL21 Rosetta DE3 were grown in lysogeny broth (LB) shaking at 37°C. Antibiotics were added to culture media at the following concentrations: ampicillin (100 μg/ml), kanamycin (30 μg/ml), and streptomycin (100 μg/ml), unless specified. All strains were stored at −80°C in LB supplemented with 10% (v/v) glycerol.

### Expression constructs

For *legA15*-deleted *L. pneumophila* strain, the *legA15* polymerase chain reaction (PCR) products using primers PL1/PL2 and PL3/PL4 were digested with Sal I/BamH I and BamH I/Sac I and inserted at Sal I/Sac I sites of pSR47s (*57*) to generate pSR47s-∆*legA15* that encodes only the first 15 and last 15 amino acids of LegA15. The full-length *legA15* was amplified using primers PL5/PL6 and inserted at Xba I/Sal I sites of plasmid pJB908 (*58*) to generate pJB908-*legA15* for *legA15* complementation.

To express LegA15 and LegA15-mCherry in mammalian cells, this gene and its fragments were amplified from *L. pneumophila* genome and inserted into vectors pCMV-3xFlag and pCMV-3xFlag-mCherry at EcoR I/Xho I sites. To express p115 in mammalian cells, this gene was amplified from a human cell complementary DNA library, digested with Bgl II/EcoR I, and inserted into vector pEGFP-c.

To produce recombinant LegA15/p115 proteins in *E. coli*, the *legA15* gene and its fragments were amplified from *L. pneumophila* genome, digested with Nhe I/Xho I, and inserted into pET28a. Similarly, the coding regions for the full-length p115 and its fragments were amplified from pEGFP-*p115*, digested with BamH I/EcoR I, and inserted into pET-GST.

All expression constructs for *legA15* point mutations or adding an N-terminal 2×FLAG were generated by our modified site-directed mutagenesis protocol with corresponding primers (*59*). All primers are listed in table S4.

### Preparation of *legA15*-deleted *L. pneumophila* strain

The *legA15* deletion plasmid pSR47s-∆*legA15* was introduced into *L. pneumophila* by a triparental mating, and transconjugants were selected on solid CYE medium with thymidine (200 μg/ml), kanamycin (20 μg/ml), and streptomycin (100 μg/ml). Bacterial mutants were screened by PCR from those grown on the medium containing 5% sucrose, as described previously (*60*). For *legA15* complementation, plasmid pJB908-*legA15* was introduced into the ∆*legA15* strain to avoid any potential variations that may arise from the thymidine auxotrophic strains (*61*). Bacteria used for infection were grown in AYE broth with streptomycin (100 μg/ml) and kanamycin (20 μg/ml) to their postexponential phase, as judged by both optical density [optical density at 600 nm (OD_600_), 3.2 to 4.0] and bacterial motility.

### Macrophage infection by *L. pneumophila*

Mouse BMDMs were seeded into Dulbecco’s modified Eagle’s medium with 10% fetal bovine serum in 24-well plates at 4 × 10^5^ and 2 × 10^5^ per well for intracellular growth and immunofluorescence assays, respectively, for 1 day before infection. U937 macrophage cells were differentiated in fresh medium by phorbol myristate acetate (10 ng/ml) for 36 hours as described previously (*62*). Cells infected with *L. pneumophila* at a multiplicity of infection (MOI) of 0.05 were synchronized at 2 hours by washing three times with fresh medium to remove all extracellular bacteria. Infected macrophages were continued to culture at 37°C in the presence of 5% CO_2_. To measure the bacterial growth, the macrophages were lysed with 0.2% saponin. Colony forming unit of infected bacteria was determined by plating a series of lysate dilutions on solid CYE medium.

### Co-immunoprecipitation

HEK293T cells were plated at a density of 2 × 10^6^ cells per 100-mm dish and transfected with plasmid pCMV-3×Flag-LegA15 using a liposomal transfection reagent (Yeasan). The cells were collected at 24 hours of post-transfection and lysed on ice for 10 min with an IP buffer [20 mM tris, 75 mM NaCl, 0.1% Triton X-100, 10% glycerol, pepstatin A (10 μg/ml), leupeptin (10 μg/ml), and 1 mM phenylmethylsulfonyl fluoride (PMSF)] by a sonication for 30 s. The cell lysis supernatant was collected by a centrifugation at 13,000 rpm for 10 min and incubated with Flag affinity resins at 4°C overnight followed by three washes with the IP buffer. The captured proteins were eluted with 50 μl of 3×Flag peptide (200 μg/ml) for 30 min on ice. The elution was boiled with an SDS loading buffer and separated on 12% SDS–polyacrylamide gel electrophoresis (SDS-PAGE) followed by immunoblotting with appropriate antibodies.

### Confocal microscopy

For intracellular LegA15 localization, HEK293T cells seeded on coverslips in a 12-well plate at a density of 1.0 × 10^4^ cells per well were transfected with pCMV-LegA15-mCherry for 18 hours. Cells were then fixed with 4% (w/v) paraformaldehyde and permeabilized with 0.1% Triton X-100 for 10 min. Organelles were stained by their specific protein antibodies (1:200). LDs were stained using dye BODIPY. Nuclei were stained by dye 4′,6-diamidino-2-phenylindole (DAPI).

For the LegA15 and p115 colocalization, HEK293T cells were cotransfected with pCMV-LegA15-mCherry and pEGFP-p115 for 18 hours. The endogenous p115 was shown by anti-p115 antibody followed by Alexa 594–conjugated secondary antibody.

For bacterial infections, Raw264.7 cells at density of 2 × 10^5^ per well were seeded on coverslips in 24-well plates and were infected with *L. pneumophila* at 0.1 MOI. After 12 hours, the cells were washed three times with phosphate-buffered saline (PBS) and fixed with 4% paraformaldehyde. The extracellular bacteria were stained with anti-*Legionella* antibody before permeabilization. Total bacteria and LegA15 were stained with the anti-*Legionella* antibody and an anti-LegA15 antibody, respectively, after the cells were permeabilized by 0.2% Triton X-100 for 10 min. The polyclonal anti-LegA15 antibody was produced by immunizing a rabbit with 6×His-LegA15 protein purified as described below. Images were acquired by a laser scanning confocal microscope (Leica, Germany). To analyze number and size of lipid droplets in cells, the cells were randomly selected and calculated in ImageJ (https://imagej.nih.gov/ij).

### Protein expression and purification

*E. coli* strain BL21/DE3 transformed with each expression construct was cultivated in LB medium at 37°C until its optical density OD_600_= 0.8 and induced by 400 μM isopropyl β-d-1-thiogalactopyranoside for 16 hours at 18°C. To prepare selenomethionine-substituted LegA15 protein, the *E. coli* expression strain grew in M9 medium supplemented with 2 mM MgSO_4_, 2 mM CaCl_2_, vitamin B (0.25 mg/ml), ampicillin (100 mg/ml), d-glucose (2 g/liter), NH_4_Cl (1 g/liter), selenomethionine (40 mg/liter), and other 19 amino acids (40 mg/liter).

6×His-tagged proteins were purified using a following protocol. The cell pellets were suspended in a lysis buffer containing 50 mM tris (pH 8), 500 mM NaCl, 10% (v/v) glycerol, 5 mM β-mercaptoethanol, protease inhibitors, and additional 20 mM imidazole and lysed using sonication on ice. The cell lysate was centrifuged for 30 min at 18,000 rpm, and its supernatant was collected and incubated with nickel sepharose beads (Sangon) for 2 hours at 4°C. The beads were washed by the lysis buffer and eluted by the lysis buffer with an additional gradient of imidazole: 50, 100, 200, and 500 mM. The best fractions that contained our protein of interest were pooled, concentrated, and further purified using a Superdex 200 size exclusion column (GE Healthcare) in 20 mM tris-HCl (pH 8.0), 150 mM NaCl, and 2 mM dithiothreitol (DTT). The best fractions of protein peaks were pooled, concentrated to 15 mg/ml with an Amicon Centrifugal filter (Millipore), flash-frozen in liquid nitrogen, and stored at −80°C.

GST-tagged p115 and its fragments were expressed using their pET-GST constructs and purified similarly as His-tagged proteins except using glutathione sepharose beads (Thermo Fisher Scientific) and captured proteins were eluted with the lysis buffer and additional 20 mM reduced glutathione. Final protein concentration was measured at absorbance at 280 nm (*A*_280_) and calculated using their theoretical extinction coefficients.

### Protein crystallization and structural determination

Crystallization of 6×His-LegA15 was performed at 25°C using a sitting drop diffusion method by mixing 0.4 μl of protein (10 mg/ml) with equal volume of reservoir solution. The crystals initially grew in 0.2 M MgCl_2_, 0.1 M bis-tris (pH 5.5), and 25% (v/v) polyethylene glycol (PEG) 3350 at 25°C in about 4 days. After optimization, the best crystallization buffer was 2 mM MgCl_2_, 0.1 M bis-tris (pH 6.2), and 22% PEG 3350. The crystals of SeMet-LegA15 were obtained under similar conditions. Complex of LegA15 and GDPNP was prepared by mixing LegA15 (10 mg/ml) and 10 mM GDPNP, and their crystals grew in 0.17 M ammonium acetate, 0.085 M sodium citrate (pH 5.6), 25.5% (v/v) PEG 4000, and 15% glycerol at 25°C in 1 week. The LegA15 crystals were cryoprotected by brief soaking in the crystallization buffer with additional 15% (v/v) PEG 200 and flash-frozen in liquid nitrogen. The LegA15/GDPNP crystals were cryoprotected by brief soaking in their crystallization buffer added with 15% glycerol and 5 mM GDPNP and flash-frozen in liquid nitrogen. Diffractions of native and SeMet-substituted protein crystals were collected at Shanghai Synchrotron Radiation Facility (SSRF) (Shanghai, China). Datasets were indexed, integrated, and scaled with HKL2000 software suite (*63*). The LegA15 structure was determined by selenium single-wavelength anomalous dispersion phasing using AutoSolve program (*64*). The LegA15/GDPNP structure was determined by molecular replacement using the LegA15 structure as a search model in Phenix (*65*). The subsequent model was manually built in Coot (*66*) and refined in Phenix program (*65*). All statistics for the data collection and structure refinement are summarized in table S2. Structural figures were generated in PyMol (http://pymol.org).

### Formaldehyde cross-linking in vivo and mass spectrometry

HEK293T cells were plated in nine 10-cm dishes at a density of 2 × 10^6^ cells per dish, six of which were transfected with pCMV-3×Flag-LegA15 plasmid, while the other three were transfected with empty pCMV-3×Flag vector as a control. The cells after 18 hours of transfection were washed twice by PBS, cross-linked with 1% (v/v) formaldehyde for 10 min at 37°C, and terminated by 125 mM glycine for 10 min at room temperature. The cells were then harvested by centrifugation at 2000 rpm for 3 min, washed twice by PBS, and lysed by sonication for 1 min in a lysis buffer [50 mM tris (pH 7.5), 150 mM NaCl, 10% glycerol, 1% NP-40, 5 mM EDTA, and 1 mM PMSF]. The cell lysate was rocked for another 4 hours at 4°C and centrifuged at 13,000 rpm for 30 min. Supernatant of the lysate was collected into a new tube and incubated with 50 μl of Flag affinity resin at 4°C overnight. The affinity resin was then collected by a centrifugation at 2000 rpm for 5 min, washed in lysis buffer three times, and boiled for 10 min with 1× SDS loading buffer. All coimmunoprecipitated proteins were separated on 12% SDS-PAGE gel and further identified by mass spectrometrical analyses.

### GST pulldown assay

GST-p115 fusion proteins of 100 μg each were incubated for 30 min with 30 μl of GST beads in a binding buffer [50 mM tris-HCl (pH 7.5) and 150 mM NaCl, 0.1% (v/v) Triton X-100, and 1 mM PMSF]. After extensive washing, the beads were further rocked overnight at 4°C with 200 μg of 6×His-LegA15 in 1 ml of the binding buffer. Unbound proteins were washed off using the buffer, and trapped proteins were eluted from the beads by 1× SDS loading buffer and resolved by 12% SDS-PAGE followed by Western blot using appropriate antibodies.

The impact of GTP/GDP on the p115 and LegA15 interaction was examined with 1 mM GTP or GDP added in the binding and washing buffers. After extensive washing in the pulldown buffer, the captured proteins were released by 1× SDS loading buffer, resolved by 12% SDS-PAGE, stained with Coomassie brilliant blue and analyzed by Western blot using appropriate antibodies.

### *p115* knockdown by shRNA

The following sequences were designed for USO1/p115 targeting shRNA: forward, 5′-GCAGCTTTGTACTATCCTAAT-CTCGAG-ATTAGGATAGTACAAAGCTGC-TTTTTGTAGAATTCTCGACCTCG-3′; and reverse: 5′-GCAGCTTTGTACTATCCTAAT-CTCGAG-ATTAGGATAGTACAAAGC-TGCCCGGTGTTTCGTCCTTTCC-3′.

The shRNA sequences were inserted into pLKO.1 lentiviral vector by “Quickgene” method (*59*), producing a plasmid pLK-p115. The lentiviral particles were packaged by cotransfecting 1.5 μg of pLK-p115 together with 1.5 μg of lentiviral packaging plasmids (pMDL:pVSV-G:pREV = 0.5:0.3:0.2) into HEK293T cells plated at 2 × 10^6^ cells each well by liposome transfection reagent Turbofect (Thermo Fisher Scientific). The cells were changed into fresh medium after 8 to 12 hours and continued to culture for another 24 to 36 hours. The viral particles were then harvested by a centrifugation at 3000 rpm for 5 min at room temperature. A new batch of HEK293T or U937 cells were infected by the lentivirus in 60-mm dishes, and continued to culture for several generations with puromycin (10 μg/ml) until a cell line was obtained. The efficiency of *p115* knockdown was evaluated by Western blot using anti-p115 antibody.

### Synchronous fluorescence titration

Synchronous fluorescence titration was performed in a binding buffer [50 mM tris-HCl (pH 7.5) and 50 mM NaCl]. LegA15 protein purified for crystallization experiments above was placed in a 2-ml quartz cuvette (10 mm by 10 mm). GTP/ATP/CTP were prepared at a concentration of 100 mM in 100 mM tris-HCl (pH 7.5) and further neutralized by 1 M NaOH. NAD^+^ was prepared at a concentration of 50 mM in 100 mM tris-HCl (pH 7.5) and further neutralized by 1 M NaOH. All nucleotides were diluted in the binding buffer to a working concentration of 20 mM. Titrations of these nucleotides to LegA15 were performed at room temperature using a Cary Eclipse fluorescence spectrophotometer (Varian, USA). The wavelength interval (Δλ) between the excitation and emission wavelengths was fixed for 15 nm. Both slit widths of excitation and emission were set to 5 nm. The ligands were titrated up to 20 μM. Relative fluorescence intensity was calculated by dividing *F*_t_ (fluorescence intensity of each titrate) to *F*_0_ (the initial density before titration).

### Isothermal titration calorimetry

The ITC experiments were performed using a microcalorimeter Affinity ITC (Waters, USA) at 20°C. The LegA15 protein purified above was diluted into a buffer of 50 mM tris-HCl (pH 8.0) and 50 mM NaCl to a final concentration of 0.1 mM. GTP and NAD^+^ stocks were prepared above and diluted in the same buffer at concentration of 1 mM. GDP was prepared as the same as GTP and used at concentration of 1 mM. Titrations were set for 20 injections of 2 μl each with 200-s intervals. Baseline subtraction and data analysis were performed using NanoAnayze. Heat spikes were integrated and fit with 1:1 binding model. The first injection of each experiment was excluded from the analysis.

### GTP hydrolysis assay

The GTP hydrolysis assay followed Mishra *et al.* (*67*) with some modifications. Briefly, 0.1 mM LegA15 purified above was loaded with 2.5 mM nucleotides in a buffer containing EDTA [50 mM tris-HCl (pH 7.5), 50 mM NaCl, 5 mM EDTA, and 1 mM DTT]. The mixture was incubated for 3 hours at room temperature and exchanged into a buffer [50 mM tris-HCl (pH 7.5) and 50 mM NaCl] by ultrafiltration to remove excess nucleotides. The concentration of nucleotide-bound LegA15 was quantified by a bicinchoninic acid protein quantification kit (Yeasan), and the bound nucleotides were assessed by the *A*_280_/*A*_260_ using the theoretical extinction coefficient of LegA15. The hydrolysis was set in a buffer [20 mM tris-HCl (pH 7.5), 50 mM NaCl, and 10 mM MgCl_2_] for 30 min at 35°C using 2.5 μM nucleotide-bound LegA15, and released phosphate was measured by a colorimetric ATPase/GTPase activity assay kit (Expedeon) according to manufacturer’s manual.

### Kinetics of GTP hydrolysis

The kinetics of GTP hydrolysis was performed as Shutes and Der (*68*) with modifications. *E. coli* phosphate binding protein (PBP; A197C) was expressed, purified by its N-terminal His tag, and labeled with 7-diethylamino-3-[*N*-(2-maleimidoethyl) carbamoyl] coumarin (MDCC; Sigma-Aldrich) to produce a sensitive phosphate probe PBP-MDCC. The reactions were set with 1 μM LegA15 purified above, 4 μM PBP-MDCC, and a GTP gradient in a buffer [20 mM tris-HCl (pH 7.5), 50 mM NaCl, and 10 mM MgCl_2_] at 35°C. The release of inorganic phosphate was monitored continuously for 30 min at an excitation wavelength of 425 nm and an emission wavelength of 457 nm. The GTP hydrolysis background was measured without LegA15 under the same condition and substracted in the final analysis. The absolute phosphate concentrations were derived from a linear curve of fluorescence using 4 μM PBP-MDCC with a concentration gradient of standard phosphate. Initial velocity (*V*_0_) was calculated by fitting to exponential model function as described by Shutes and Der (*68*). In addition, the enzyme parameters such as *K*_m_ and *V*_max_ were derived by fitting the measurements to the Michaelis-Menten equation.

### Cell viability assay

Cell viability assay was performed by cell counting kit-8 (Yeasan). Mammalian cells were plated in a 96-well plate at a density of 5 × 10^3^ cells per well and transfected with the LegA15 expression construct. After 24-hour transfection, each well was added with 10% formazan and incubated for 2 hours at 37°C. The absorbance of the wells at 450 nm was measured and repeated at least three times.

### Golgi fragmentation analysis

Fragmented Golgi was defined according to Joshi *et al.* (*69*). Cells were fixed as described above and stained with anti-syntaxin 6 antibody for Golgi apparatus and dye DAPI for nucleus. Cells with scattered dots in the perinuclear region or isolated dots (mini-Golgi) dissociated from the major Golgi apparatus were counted as those with fragmented Golgi. More than 300 cells were collected and examined for each transfection and counted in ImageJ.

### SEAP assay

SEAP assay was performed by an SEAP reporter assay kit (Invivogen). HEK293T cells were cultured in 96-well plates at a density of 1 × 10^4^ cells per well and transfected with the LegA15 expression construct. Its empty vector was used as a control. The cells and culture medium were separated at 24 hours of transfection by a centrifugation at 10,000 rpm for 5 min. The culture medium supernatant was transferred to a new tube and heated at 65°C for 10 min to inhibit endogenous alkaline phosphatases. The cells were lysed by radioimmunoprecipitation assay lysis buffer (Thermo Fisher Scientific) on ice for 30 min and centrifuged at 10,000 rpm for 5 min. In addition, their supernatant was collected into a new tube and also heated at 65°C for 10 min. The SEAP activity was detected at 410 nm according to the manufacturer’s manual and repeated at least three times.

### Data analysis

Pearson correlation coefficient (*r*) for colocalization analysis and significant difference analysis of one-way analysis of variance (ANOVA) and Student’s *t* test were calculated all in GraphPad Prism 8 software (San Diego, USA). *P* < 0.05 was considered as significant difference.

### Materials resources

Resources of key materials in this study are listed in table S5.

## Supplementary Materials

### Other Supplementary Material for this manuscript includes the following:

Raw data S1 to S19

## Notes

View/request a protocol for this paper from *Bio-protocol*.
