# Supplementary material for: Atypical Legionella GTPase effector hijacks host vesicular transport factor p115 to regulate host lipid droplet
Source: Sci Adv. 2022 Dec 16;8(50):eadd7945. doi: 10.1126/sciadv.add7945 (PMC9757750; doi:10.1126/sciadv.add7945)
Supplement: Supplementary file 1 — Figs. S1 to S11 Tables S1 to S5 [file sciadv.add7945_sm.pdf]

Science Advances
AAAS

Supplementary Materials for

Atypical *Legionella* GTPase effector hijacks host vesicular transport factor p115 to regulate host lipid droplet

Tao-Tao Chen *et al.*

Corresponding author: Aidong Han, ahan@xmu.edu.cn; Zhao-Qing Luo, luoz@purdue.edu

Sci. Adv. **8**, eadd7945 (2022)
DOI: 10.1126/sciadv.add7945

This PDF file includes:

Figs. S1 to S11
Tables S1 to S5
Legends for raw data S1 to S19

Other Supplementary Materials for this manuscript includes the following:

Raw data S1 to S19

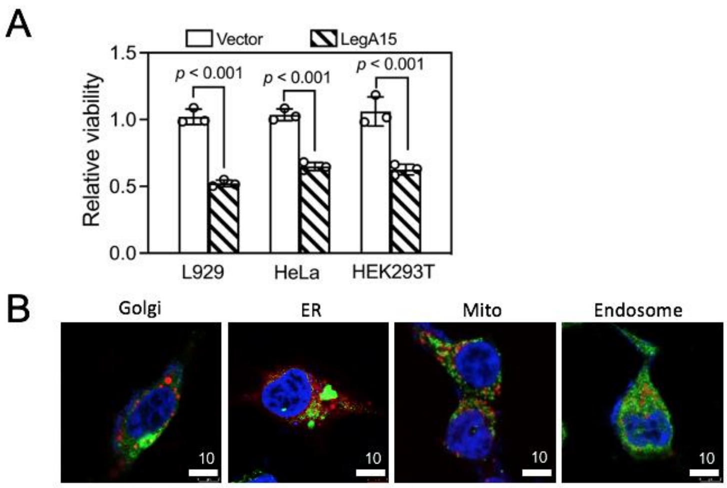

Fig. S1. Cytotoxicity and localization of over-expressed LegA15. (A) Viability of mammalian cells expressing FLAG-LegA15 for 24 h. These cells transfected with the empty LegA15 expression vector were used as negative controls. The results shown were from three independent experiments with five replicates, and analyzed by one-way ANOVA assay. (**B)** Ectopically expressed LegA15-mCherry does not co-localize with Golgi, endoplasmic reticulum (ER), mitochondria (Mito) or endosome. The organella in green were labeled by specific antibodies for biomarkers TGN46, ERp57, AIF, and Rab7, respectively; Nuclei were stained by DAPI in blue. Scale bars: µm.

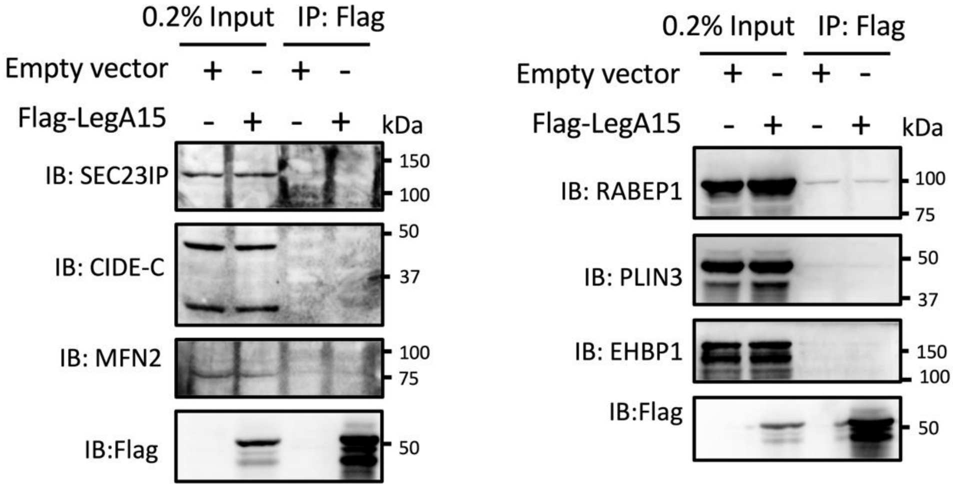

Fig. S2. Other proteins failed to show their interaction with LegA15. HEK293T cells expressing Flag-LegA15 were immunoprecipitated by anti-Flag antibody, and potential interaction proteins were detected by their specific antibodies. Cells transfected with an empty vector were used as a negative control.

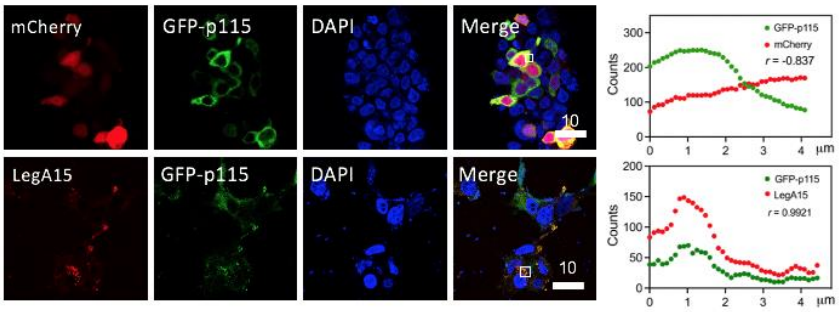

Fig. S3. LegA15 co-localizes with over-expressed p115 in HEK293T cells. The cells expressing both LegA15-mCherry and GFP-p115 were fixed and imaged by a confocal microscope. Shown on the right were co-localization analyses with Pearson correlation (*r*) on ROI in white boxes. X-axis is width of ROI and Y-axis is pixel counts for the fluorescence intensity of LegA15 (red curve) and p115 (green curve). Nuclei were stained by DAPI in blue. Scale bars: µm.

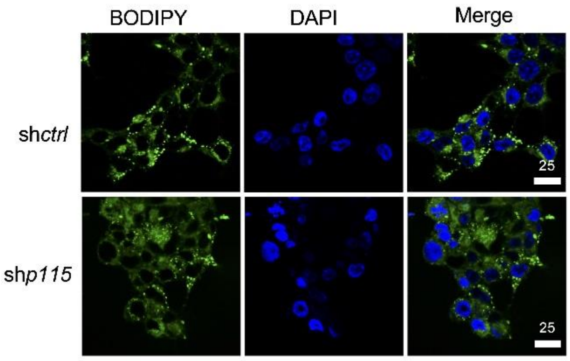

Fig. S4. The *p115* knockdown did not appear to affect cellular LDs in HEK293T cells. LDs were stained with BODIPY in green. Nuclei were stained by DAPI in blue. Images were acquired using a confocal microscope. Scale bars: µm.

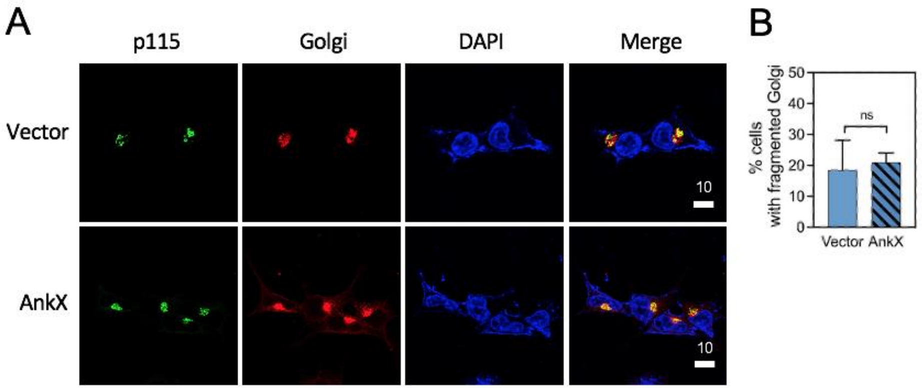

Fig. S5. AnkX does not induce Golgi fragmentation. HEK293T cells were over-expressed with Flag-AnkX for 24 h. p115 shown in green was blotted by anti-p115 antibody, and Golgi shown in red was blotted by anti-STX6 antibody. Cells transfected with an empty vector were used as a negative control. Quantification of cells with fragmented Golgi (**B**) was averaged from three groups of cells with more than 100 randomly selected cells in each group.

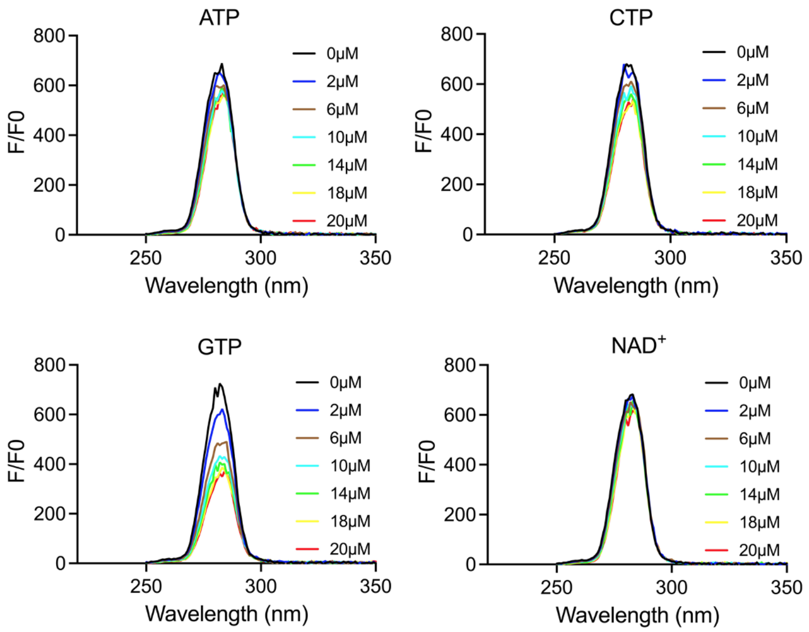

Fig. S6. Fluorescence titrations for nucleotides binding to LegA15. Details of titrations were described in method section.

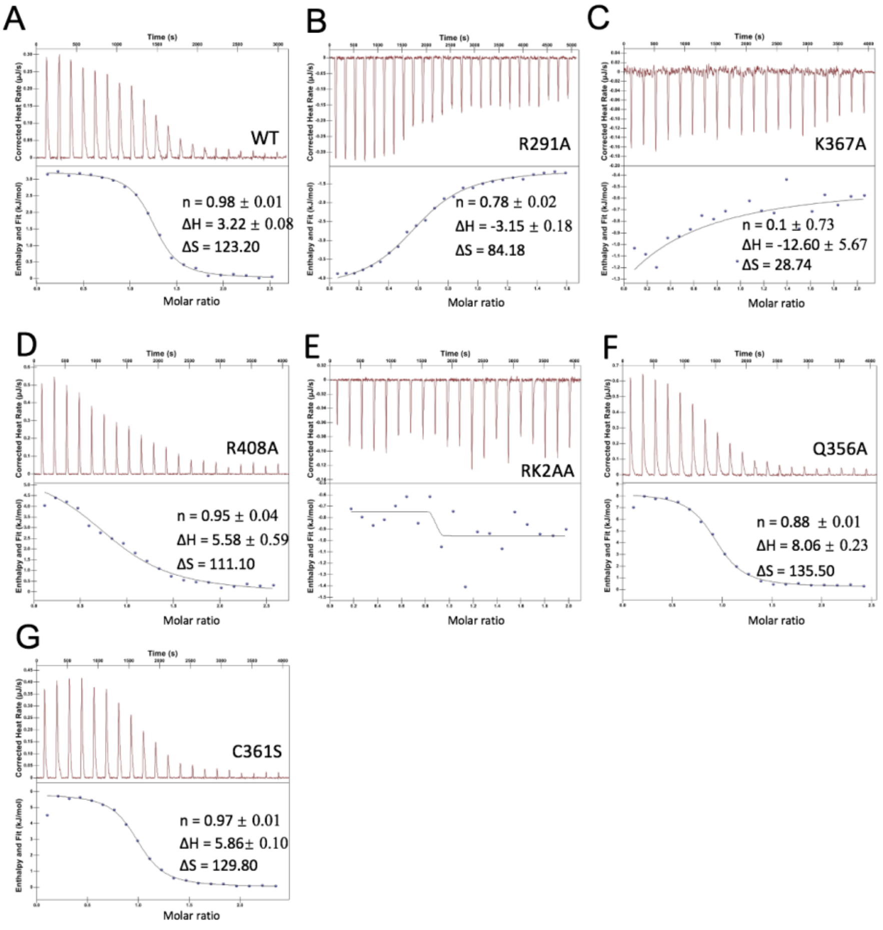

Fig. S7. Mutational analyses of the GTP-binding pocket of LegA15. (A-G) Original ITC data for quantification of GTP binding with LegA15 and its mutants in the GTP binding pocket. The derived parameters are shown within.

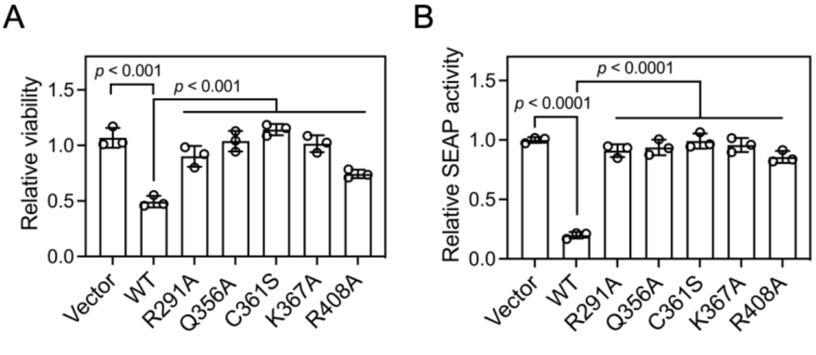

Fig. S8. The GTP-binding pocket of LegA15 is important for its cytotoxicity. (**A**) Viability of HEK293T cells when Flag-LegA15 or its mutants were expressed for 36 h. (**B**) SEAP activity of HEK293T cells when Flag-LegA15 or its mutants were expressed

for 24 h. Cells transfected with its empty vector were used as a negative control. Results shown were from three independent experiments with five replicates, and analyzed by one-way ANOVA assay.

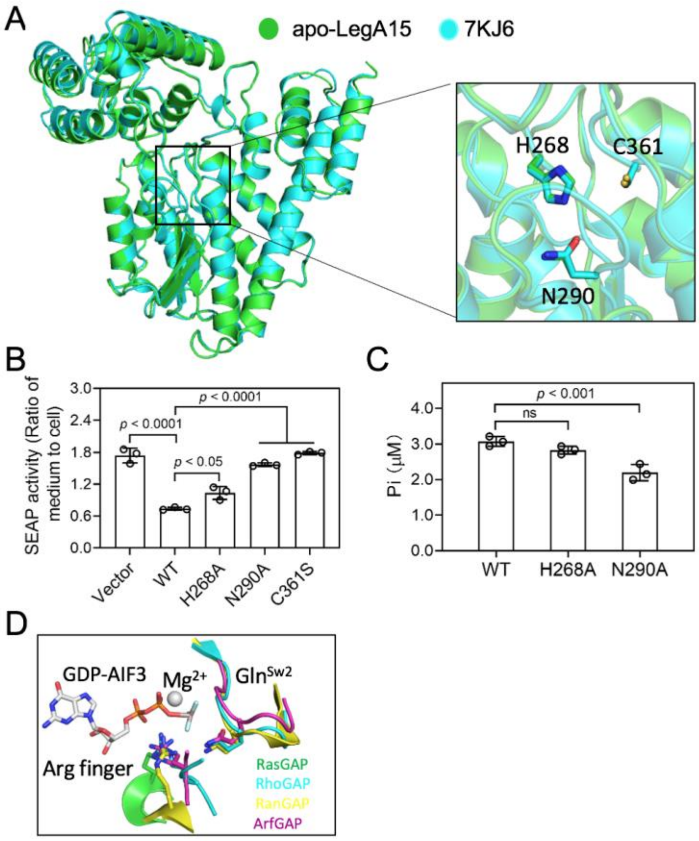

Fig.S9. Residues His268 and Asn290 are not critical sites to LegA15. (A) Structural alignment of LegA15. Structures of LegA15 are colored in green (this work) and cyan (PDB ID: 7KJ6) with a proposed catalytic triad C361-H268-N290 shown in a close-up view. (**B, C**) Mutations of the triad residues affected LegA15 in SEAP activity when over-expressed (B) and its GTP hydrolysis (C). HEK293T cells transfected with its vector were used as a negative control. The SEAP activity was shown by ratio of culture supernatant to the whole cell. Data presented a mean from three independent experiments in triplicate and analyzed by one-way ANOVA assay. (**D**) Comparison of Lys367 of LegA15 with conventional arginine finger for GTP hydrolysis. The arginine fingers in representative GTPase structures of RasGAP (PDB code: 1WQ1), ArfGAP

(PDB code: 3BH7), RhoGAP (1OW3), and RanGAP (1K5D) are super-imposed. The conserved glutamine and arginine residues are shown as stick models.

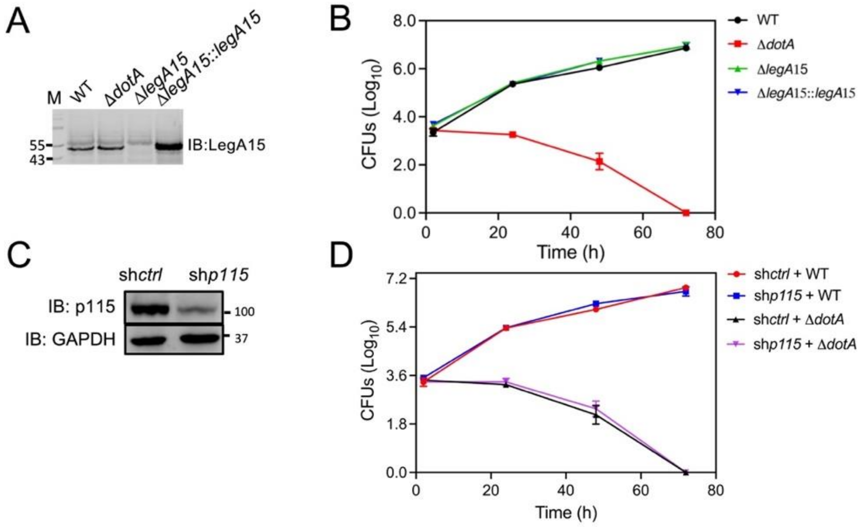

Fig. S10. Neither lack of LegA15 nor knockdown of *p115* affects intracellular growth of *L. pneumophila.* (**A, B)** *L. pneumophila* strains with LegA15 deletion Δ*legA15* and its complementation Δ*legA15:plegA15* in wild-typed U937 cells. The LegA15 level in the saponin-solublized fractions of the U937 cells infected by these bacteria was detected with anti-LegA15 antibody (A). The growth curves of these bacteria in the U937 cells represent an average of three independent experiments (B). (**C, D**) *L. pneumophila* grew in *p115* knockdown U937 cells. The U937 cells were infected with lentivirus expressing shRNA against *p115* (sh*p115*) or scrambled shRNA (sh*ctrl*). The level of p115 was detected by anti-p115 antibody (C). The growth curves of these bacteria in the *p115* knockdown U937 cells represent an average of three independent experiments (D). *L. pneumophila* Δ*dotA* was used for a negative control.

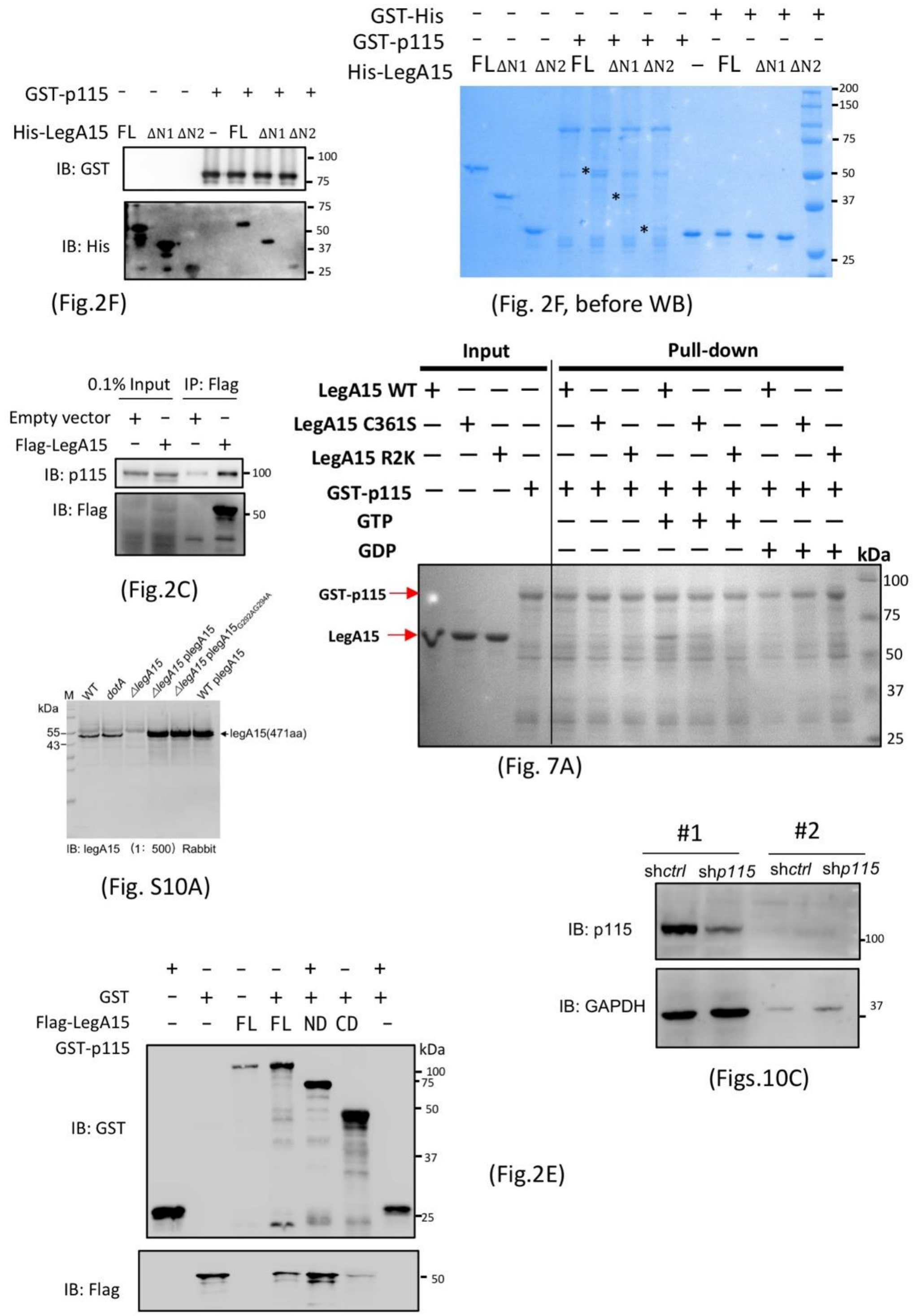

Fig. S11. Original key data. All data with associated figures are arranged according to their appearance in the paper.

Table S1. Proteins in the vesicular trafficking and membrane fusion co-immunoprecipitated with LegA15 and identified by mass spectrometry.

No.	Name	Accession	-10lgP	Coverage (%)	Unique	Description
1	USO1	O60763	403.71	46	62	General vesicular transport factor p115
2	IQGA1	P46940	383.45	45	60	Ras GTPase-activating-like protein IQGAP1
3	COPG1	Q9Y678	377.33	44	39	Coatomer subunit gamma
4	COPB	P53618	376.89	45	50	Coatomer subunit beta
5	COPA	P53621	376.56	43	54	Coatomer subunit alpha
6	ESYT1	Q9BSJ8	296.72	27	24	Extended synaptotagmin
7	S23IP	Q9Y6Y8	294.03	21	27	SEC23-interacting protein
8	F5H7S7	F5H7S7	282.75	26	30	Ras GTPase-activating-like protein IQGAP2
9	VPS35	Q96QK1	277.42	28	17	Vacuolar protein sorting-associated protein 35
10	GDIB	P50395	275.16	44	12	Rab GDP dissociation inhibitor beta
11	COPG2	Q9UBF2	264.2	23	12	Coatomer subunit gamma
12	ACSL3	O95573|	262.28	28	14	Long-chain-fatty-acid--CoA ligase
13	RBGPR	Q9H2M9	260.83	11	16	Rab3 GTPase-activating protein non-catalytic subunit
14	AP2B1	P63010	257.11	28	11	AP-2 complex subunit beta
15	RAE2	P26374	255.52	26	17	Rab proteins geranylgeranyltransferase component A2
16	EXOC4	Q96A65	250.8	24	18	Exocyst complex component
17	RAGP1	P46060	247.34	32	18	Ran GTPase-activating protein
18	SC24C	P53992	242.55	19	17	Protein transport protein Sec24C
19	RHG17	Q68EM7	242.36	14	10	Rho GTPase-activating protein 17
20	SC24A	O95486	238.09	18	13	Protein transport protein Sec24A
21	LLGL1	Q15334	236.71	17	13	Lethal(2) giant larvae protein homolog
22	TBCD4	O60343	235.98	12	12	TBC1 domain family member
23	AP3B1	O00203	231.97	14	11	AP-3 complex subunit beta
24	WDR11	Q9BZH6	222.17	16	14	WD repeat-containing protein
25	TBC9B	Q66K14	218.56	9	11	TBC1 domain family member 9B
26	LRBA	P50851	218.18	7	16	Lipopolysaccharide-responsive and beige-like anchor protein
27	VP13A	Q96RL7	216.25	6	13	Vacuolar protein sorting-associated protein 13A
28	VP13C	Q709C8	212.36	8	17	Vacuolar protein sorting-associated protein 13C
29	RBGP1	Q9Y3P9	209.43	13	10	Rab GTPase-activating protein
30	WDR44	Q5JSH3	204.7	17	11	WD repeat-containing protein 44
31	RBP1	Q15311	203.73	17	11	RalA-binding protein
32	RABE1	Q15276	202.05	18	13	Rab GTPase-binding effector protein
33	EXOC2	Q96KP1	197.91	16	10	Exocyst complex component
34	FAF2	Q96CS3	197.63	21	9	FAS-associated factor
35	VP26A	O75436	197.51	32	7	Vacuolar protein sorting-associated protein 26A
36	EXOC1	Q9NV70	191.97	17	9	Exocyst complex component
37	SRGP2	O75044	171.28	12	9	SLIT-ROBO Rho GTPase-activating protein
38	RANG	P43487	167.73	27	8	Ran-specific GTPase-activating protein
39	RASA1	P20936	166.58	12	8	Ras GTPase-activating protein
40	AP1M1	Q9BXS5	160.8	24	8	AP-1 complex subunit mu-1
41	ATG9A	Q7Z3C6	158.78	8	5	Autophagy-related protein 9A
42	RBG1L	Q5R372	158.61	26	3	Rab GTPase-activating protein 1-like
43	AP1G1	O43747	157.81	11	7	AP-1 complex subunit gamma
44	SNX17	Q15036	151.18	19	6	Sorting nexin-17
45	TXLNA	P40222	151.04	14	5	Alpha-taxilin
46	SCRIB	Q14160	150.12	10	11	Protein scribble homolog

47	COPZ1	P61923	148.26	36	4	Coatomer subunit zeta
48	TPPC8	Q9Y2L5	147.63	9	8	Trafficking protein particle complex subunit
49	MFN2	O95140	147.11	11	6	Mitofusin
50	ACAP2	Q15057	143.23	7	5	Arf-GAP with coiled-coil ANK repeat and PH domain-containing protein 2
51	ARAP1	Q96P48	141.45	10	9	Arf-GAP with Rho-GAP domain ANK repeat and PH domain-containing protein
52	TPC11	Q7Z392	139.23	6	6	Trafficking protein particle complex subunit
53	NSF	P46459	139.13	11	8	Vesicle-fusing ATPase
54	RBG1L	Q5R372	138.63	8	3	Rab GTPase-activating protein 1-like
55	NGAP	Q9UJF2	137.54	6	6	Ras GTPase-activating protein nGAP
56	AP2S1	P53680	135.27	33	4	AP-2 complex subunit sigma
57	EHBP1	Q8NDI1	130.13	5	4	EH domain-binding protein
58	SNX2	O60749	127.84	11	4	Sorting nexin-2
59	MFN1	Q8IWA4	126.56	9	5	Mitofusin
60	SCFD1	Q8WVM8	125.92	14	6	Sec1 family domain-containing protein
61	VPS51	Q9UID3	125.13	8	4	Vacuolar protein sorting-associated protein 51 homolog
62	RB3GP	Q15042	123.97	8	6	Rab3 GTPase-activating protein catalytic subunit
63	GAPD1	Q14C86	121.82	7	8	GTPase-activating protein and VPS9 domain-containing protein
64	RLGPB	Q86X10	121.03	4	4	Ral GTPase-activating protein subunit beta
65	RHG35	Q9NRY4	119.69	5	5	Rho GTPase-activating protein 35
66	VP26B	Q4G0F5	119.04	19	5	Vacuolar protein sorting-associated protein 26B
67	RHG05	Q13017	117.3	4	5	Rho GTPase-activating protein 5
68	M2OM	Q02978	114.9	20	3	Mitochondrial 2-oxoglutarate/malate carrier protein
69	TBCD1	Q86TI0	114.21	15	1	TBC1 domain family member 15
70	FCHO2	QUJRZ9	110.6	14	3	F-BAR domain only protein
71	ZFY16	Q7Z3T8	109.71	4	4	Zinc finger FYVE domain-containing protein 16
72	SNX5	Q9Y5X3	109.1	11	4	Sorting nexin-5
73	SNAA	P54920	105.97	23	4	Alpha-soluble NSF attachment protein
74	MILK1	Q8N3F8	105.62	6	4	MICAL-like protein
75	TBC15	Q8TC07	104.85	6	2	TBC1 domain family member 15
76	C2CD5	Q86YS7	104.27	4	3	C2 domain-containing protein 5
77	VPS36	Q86VN1	104.17	15	4	Vacuolar protein-sorting-associated protein 36
78	VTA1	Q9NP79	103.47	14	3	Vacuolar protein sorting-associated protein VTA1 homolog
79	G3BP1	Q13283	101.81	9	3	Ras GTPase-activating protein-binding protein
80	ANR50	Q9ULJ7	99.48	3	3	Ankyrin repeat domain-containing protein 50
81	VAT1	Q99536	96.56	16	5	Synaptic vesicle membrane protein VAT-1 homolog
82	IQGA3	Q86VI3	94.98	4	3	Ras GTPase-activating-like protein IQGAP3
83	ARF5	P84085	94.62	28	1	ADP-ribosylation factor 5
84	AUP1	Q9Y679	92.95	10	3	Ancient ubiquitous protein
85	SYNJ1	O43426	91.49	3	3	Synaptojanin
86	SCAM	O15126	90.98	12	2	Secretory carrier-associated membrane protein
87	PTN23	Q9H3S7	90.88	3	4	Tyrosine-protein phosphatase non-receptor type 23
88	PLIN3	O60664	87.43	6	3	Perilipin-3
89	VAPB	O95292	82.46	11	2	Vesicle-associated membrane protein-associated protein B/C
90	ASAP1	Q9ULH1	69.48	3	2	Arf-GAP with SH3 domain ANK repeat and PH domain-containing protein
91	RGPA1	Q6GYQ0	68.69	2	2	Ral GTPase-activating protein subunit alpha
92	PLIN1	O60240	30.05	1	1	Perilipin-1
93	SNX27	Q96L92	29.79	3	1	Sorting nexin-27

Table S2. Data collection and refinement statistics.

	**LegA15** (PDB ID: 7EW8)	**LegA15/GDPNP** (PDB ID: 7XQL)
**Data Collection**		
Space group	$P2_1$	$P2_12_12_1$
**Cell dimensions**		
a,b,c (Å)	66.058, 75.997, 104.814	75.606, 77.833, 170.656
α,β,γ (◦)	90.000, 90.807, 90.000	90.000, 90.000, 90.000
Wavelength (Å)	0.9789	0.9792
Resolution (Å)	40 -2.59 (2.63 - 2.59)	34.6 - 2.27 (2.353 - 2.27)
No. reflections	206532 (32235)	627872 (35047)
R_{merge}	0.091 (0.565)	0.119 (0.545)
I/σ	21.77 (2.55)	18.55 (6.3)
$CC_{1/2}$	0.995 (0.851)	0.998 (0.854)
R_{pim}	0.03 (0.274)	0.035 (0.096)
R_{meas}	0.093 (0.628)	0.128 (0.595)
Completeness (%)	99.6 (94.9)	99.10 (95.08)
Redundancy	6.4 (4.5)	12.8 (11.5)
**Refinement**		
Resolution (Å)	29.20-2.59 (2.68 - 2.59)	34.6 - 2.27 (2.353 - 2.27)
No. reflections	31295 (3011)	46801 (4426)
R_{work}/R_{free} (%)	20.86/23.83 (34.41/38.67)	18.22/22.78 (21.81/25.99)
**No. atoms**		
Protein	6741	6872
Ligand	0	32
Solvent	176	217
B factors (Å^2)	47.37	42.60
Protein	47.77	42.49
Solvent	32.29	41.81
**R.m.s deviations**		
Bond Lengths (Å)	0.006	0.008
Bond angles (◦)	0.89	0.91
**Ramachandran plot (%)**		
Favored	95.82	97.73
Allowed	4.18	2.27
Outliers	0.00	0.00

Note: Statistics for the highest resolution shell are shown in parentheses.

Table S3. Top structures similar to LegA15 analyzed by PDBeFold server.

	Z-Score	RMSD (Å)	Seq (id%)	Match (PDB_ID)	Description
1	2.5	4.65	8	5mpa:A	26S proteasome in presence of ATP (S2)
2	2.3	4.45	10	1esq:A	Thiazole kinase mutant (C198S) with ATP and thiazole phoshate
3	2.3	4.67	8	4g1c:B	Human SIRT5 bound to succ-IDH2 and carba-NAD
4	2.1	4.27	6	4utn:A	Zebra fish Sirtuin 5 in complex with succinylated CPS1-peptide
5	1.3	5.09	10	1i7e:A	R. Rubrum transhydrogenase domain I with bound NADH
6	1.3	5.01	8	6cwa:B	PHGDH in complex with NADH and 3-phoshoglycerate
7	1.2	5.45	5	1gzg:B	Mg^{2+}-dependent porphobilinogen synthase from Pseudomonas Aeruginosa with 5-fluoolevulinic acid
8	1.1	4.84	4	1mlv:A	A SET domain protein methyltransferase
9	1.0	5.17	4	4mag:A	The periplasmic sialic acid binding protein from Vibrio Cholerea
10	1.0	4.73	7	3zgo:C	Human SIRT2 apo form

Table S4. Primers used in this study

Primers	Sequences	Usage
*legA15*KO-up-F (PL1)	ctggagctcagtcattatctcgaagcctag	To construct Δ*legA15*
*legA15*KO-up-R (PL2)	tgctttcagatttatcggtggttgaaatttttg	To construct Δ*legA15*
*legA15*KO-down-F (PL3)	caccgataaatctgaaagcagtatgacact	To construct Δ*legA15*
*legA15*KO-down-RI (PL4)	acgcgtcgacatggtagatggcttattacaatatg	To construct Δ*legA15*
legA15-XbaI-F (PL5)	agc**TCTAGA**atgttgactcctccg	To clone *legA15* into pJB908
legA15-SalI-F (PL6)	agc**GTCGAC**ttaaatggttttccctgg	To clone *legA15* into pJB908
legA15-NheI-F (PL7)	agc**GCTAGC**atgttgactcctccg	To build pET28-*legA15*
legA15-XhoI-R (PL8)	agc**CTCGAG**ttaaatggttttccctgg	To build pET28-*legA15*
legA15-EcoRIgt-F (PL9)	ccg**GAATTC**GTatgttgactcctcc	To build pCMV-*legA15*
legA15-*mCherry*-F	gaaaaccattctcgagatggtgagc	For gene fusion
legA15-*mCherry*-R	ctcgagaatggttttccctggttgg	For gene fusion
legA15$_{(1\text{-}192)}$-*mCherry*-Xho1-R	gcccttgctcaccatctcgagcttactgtcaagagcaa	To build *legA15 fragment*
legA15$_{(95\text{-}471)}$-*mCherry* -F	agcgctccattggatgacagcgcacaaagcgcattaattca agccacaaact	To build *legA15 fragment*
legA15$_{(95\text{-}471)}$-*mCherry* -R	tgtgcgctgtcatccaatggagcgcttaatttatccaaattttat cg	
legA15$_{(95\text{-}471)}$ -NheI	agc**GCTAGC**cacagcgcacaaagcgcattaattc	To build *legA15 fragment*
legA15$_{(195\text{-}471)}$ -NheI	agc**GCTAGCcccacttatcaattgatgacagattc**	To build *legA15 fragment*
legA15-*FLAG*-F	catcatcatcatcacgattacaaggacagcgatgacaag	To insert *FLAG* into pET28-*legA15*
legA15-*FLAG*-R	cggcaccaggccgctgctcttgtcatcgtcgtccttgtaatc	To insert *FLAG* into pET28-*legA15*
mCherry-NotI-R	agc**GCGGCCGC**ttacttgtacagctcgtccatgc	To clone *mCherry*
legA15-G292A/G294A-F	cgtgcacttgcagcaaagagcagc	For mutagenesis
legA15-G292A/G294A-R	gctgcaagtgcacgattggtatatacc	For mutagenesis
legA15-K296A/S297A-F	gagcagcagcaagcgagcatgg	For mutagenesis
legA15-K296A/G297A-R	cgcttcctgctgctccaagacc	For mutagenesis
legA15-R291A-F	ccaatgccggtcttggagcaaacagc	For mutagenesis
legA15-R291A-R	ccaagaccggcattggtatataccaggtagcc	For mutagenesis
legA15-K367A-F	ccaactccgcctccaacatagagggaatac	For mutagenesis
legA15-K367A-R	gttggaggcggagttggctattgtgc	For mutagenesis
legA15-R408A-F	caaaacatatggccgttgaaaaagtcaatgaattagcc	For mutagenesis
legA15-R408A-R	ttttcaacggccatatgttttgtaaattccttgtattc	For mutagenesis
legA15-Q356A-F	caaaaaggagccaaaaatgacaattgcacaatagcc	For mutagenesis
legA15-Q356A-R	catttttggctcctttttgtttgatatggtgaatgg	For mutagenesis
legA15-C361S-F	gacaattccacaatagccaactcc	For mutagenesis
legA15-C361S-R	gctattgtggaattgtcatttttttgtcc	For mutagenesis
p115$_{1\text{-}651}$-BamHI-F	agc**GGATCC**atgaatttcctccgcgggg	To build *p115* fragment
p115$_{1\text{-}651}$-EcoRI-R	agc**GAATT**ccacaatattgtcatgctgttctaatgttttt	To build *p115* fragment
p115$_{651\text{-}961}$-BamHI-F	agc**GGATCC**attgtgactcactacaaaaatatg	To build *p115* fragment
p115$_{651\text{-}961}$-EcoRI-R	agc**GAATTC**ctagatatgatctagatccttgcc	To build *p115* fragment

Note: The restriction sites are in bold uppercase.

Table S5. Resources of key materials used for this study

REAGENT or RESOURCE	SOURCE	IDENTIFIER
Antibodies		
Rabbit polyclonal anti-Flag	Proteintech, China	20543-1-AP
Rabbit polyclonal Anti-HA	Proteintech, China	51064-2-AP
Rabbit polyclonal Anti-p115	Proteintech, China	13509-1-AP
Rabbit polyclonal Anti-LegA15	This study	N/A
Rabbit polyclonal Anti-*Legionella*	Abcam, England	ab20943
Rabbit polyclonal Anti-PLIN3	Proteintech, China	10694-1-AP
Rabbit polyclonal Anti-CIDE-C	Proteintech, China	12287-1-AP
Rabbit polyclonal Anti-GST	Proteintech, China	10000-0-AP
Rabbit polyclonal Anti-Rab7	Proteintech, China	15967-1-AP
Mouse polyclonal Anti-p115	Proteintech, China	68100-1-Ig
Mouse monoclonal Anti-His	Proteintech, China	66005-1-Ig
Rabbit polyclonal Anti-EHBP1	Proteintech, China	17637-1-AP
Rabbit polyclonal Anti-RABEP1	Proteintech, China	14350-1-AP
Rabbit polyclonal Anti-MFN2	Proteintech, China	12186-1-AP
Rabbit polyclonal Anti-SEC32IP	Proteintech, China	20892-1-AP
Rabbit polyclonal Anti-ERp57/ERp60	Proteintech, China	15967-1-AP
Rabbit polyclonal Anti-AIF	Proteintech, China	17984-1-AP
Rabbit polyclonal Anti-Syntaxin 6	Proteintech, China	10841-1-AP
HRP-conjugated Goat anti-Mouse	Proteintech, China	SA00001-1
HRP-conjugated Goat anti-Rabbit	Proteintech, China	SA00001-2
CoraLite488-Goat anti-Rabbit	Proteintech, China	SA00013-2
CoraLite488-Goat anti-Mouse	Proteintech, China	SA00013-1
CoraLite594-Goat anti-Rabbit	Proteintech, China	SA00013-4
Bacterial strains		
Legionella pneumophila Philadelphia-1 strain Lp02	Ref. 57	N/A
L.pneumophila Lp03 Δ*dotA*	Ref. 58	N/A
L.pneumophila Δ*legA15*	This study	N/A
L.pneumophila Δ*legA15::legA15*	This study	N/A
Escherichia coli DH5α	Tangen, China	N/A
Escherichia coli BL21/DE3	Tangen, China	N/A
Chemicals and peptides		
Dulbecco’s Modified Eagle’s Medium with L-gluatamine and sodium pyruvate	Gibco, USA	C11995500BT
RIPM1640 medium	Gibco, USA	C11875500BT
Fetal bovine serum	Gibco, USA	10270-106
3xFLAG peptide	Genescript, China	RP10586
Formaldehyde (37%)	Sigma, USA	F8775
GTP	Sigma, USA	10106399001
GDP	Sigma, USA	G7127
GDPNP	GlpBio	GC13248-10
NAD$^+$	Sigma, USA	53-84-9
ATP	Sigma, USA	A1852
CTP	Sangon, China	A620134
BODIPY	Invivogen	D3922
TurboFect^{TM} transfection reagent	Thermo, USA	R0531
Anti-DYKDDDDK G1 affinity resin	GenScript, China	L00432-1
Glutathione agarose resin	Thermo, USA	16101

Nickel affinity agarose	Sangon, China	C60033
Critical commercial assays		
SEAP Reporter Assay Kit	Invivogen, China	Psetz-seap
High Throughput Colorimetric ATPase Assay Kit	Expedeon, UK	303−0030
Cell Counting Kit-8	Yeasan, China	K1018
Deposited data		
LegA15 structure	Protein Data Bank	7ew8
LegA15/GDPNP structure	Protein Data Bank	7xql
Cell lines		
HEK293T cells	Li lab (Xiamen University)	N/A
HEK293T-*p115* KD cells	This study	N/A
U937 cells	ATCC, USA	N/A
U937-*p115* KD cells	This study	N/A
BMDM cells	ATCC, USA	N/A
Raw264.7	ATCC, USA	N/A
HeLa	Li lab (Xiamen University)	N/A
L929	Han lab (Xiamen University)	N/A
Oligonucleotides		
Primers used in this study are listed in Table S4	This study	N/A
Constructs		
pET28-*legA15*	This study	N/A
pET28-2xFLAG-*legA15*	This study	N/A
pET28-*legA15*^{R291A}	This study	N/A
pET28-*legA15*^{Q356A}	This study	N/A
pET28-*legA15*^{C361S}	This study	N/A
pET28-*legA15*^{K367A}	This study	N/A
pET28-*legA15*^{R408A}	This study	N/A
pET28-*legA15*$^{R291A/K367A/R408A}$	This study	N/A
pET28-*legA15*^{K367R}	This study	N/A
pET28-*legA15*^{H268A}	This study	N/A
pET28-*legA15*^{N290A}	This study	N/A
pET28-*legA15 95-471*	This study	N/A
pET28-*legA15 195-471*	This study	N/A
pCMV-3xFLAG-*legA15*	This study	N/A
pCMV-3xFLAG-*legA15*^{R291A}	This study	N/A
pCMV-3xFLAG-*legA15*^{Q356A}	This study	N/A
pCMV-3xFLAG-*legA15*^{C361S}	This study	N/A
pCMV-3xFLAG-*legA15*^{K371A}	This study	N/A
pCMV-3xFLAG-*legA15*^{R408A}	This study	N/A
pCMV-3xFLAG-*legA15*$^{R291A/K367A/R408A}$	This study	N/A
pCMV-3xFLAG-*legA15-mCherry*	This study	N/A
pCMV-3xFLAG-*legA15 (1-192) -mCherry*	This study	N/A
pCMV-3xFLAG-*legA15 (95-471) -mCherry*	This study	N/A
pCMV-3xFLAG-*mCherry*	This study	N/A
pCMV-3xFLAG-*legA15*^{C361S}-*mCherry*	This study	N/A
pCMV-3xFLAG-*legA15*$^{R291A/K367A/R408A}$-*mCherry*	This study	N/A
pGEX6p-*p115*	This study	N/A
pGEX6p-*p115* 1-651	This study	N/A
pGEX6p-*p115* 651-821	This study	N/A
pEGFP-*p115* (For GFP-p115 fusion)	This study	N/A
pSR47s-Δ*legA15* (5’ 45bp + 3’ 45bp)	This study	N/A

pJB908-*legA15*	This study	N/A
pJB908-2xFYVE-GFP	This study	N/A
pLKO.1-sh*p115*	This study	N/A
Others		
ImageJ	NIH	N/A
Graghpad Prism 7.0	San Diego, USA	N/A
Pymol	DeLano Scientific LLC	N/A

Raw auxiliary data captions:

Raw_data_1: Fig.2H Knockdown of p115 suppresses the cytotoxicity of LegA15.

Raw_data_2: Fig.3C LegA15 inhibits the secretion of SEAP.

Raw_data_3: Fig.4B Ligand binding analyses of LegA15 by synchronous fluorescence titration.

Raw_data_4: Fig.5A The GTPase activity of LegA15.

Raw_data_5: Fig.5B Enzyme kinetics of LegA15 measured using a concentration gradient of GTP with 1 μM LegA15.

Raw_data_6: Fig.5C The divalent metal ions were required for the GTPase activity.

Raw_data_7: Fig.5D The GTPase activity of LegA15 was impaired by all GTP binding mutations.

Raw_data_8: Fig.5F Replacement of Lys367 with arginine preserved the GTPase activity of LegA15.

Raw_data_9: fig. S1A Viability of mammalian cells expressing FLAG-LegA15.

Raw_data_10-13: fig. S6 Fluorescence titrations for nucleotides binding to LegA15.

Raw_data_14: fig. S8A Viability of HEK293T cells expressing Flag-LegA15 or its mutants.

Raw_data_15: fig. S8B SEAP activity expressing Flag-LegA15 or its mutants.

Raw_data_16: fig. S9B Mutations of the triad residues affected LegA15 in SEAP activity.

Raw_data_17: fig. S9C Mutations of the triad residues affected LegA15 in GTP hydrolysis.

Raw_data_18: fig. S10B Impact of *legA15* on the growth of *L. pneumophila* in the U937 cells.

Raw_data_19: fig. S10C Impact of *p115* on the growth of *L. pneumophila* in the U937 cells
